# Supplementary figures and images for: Effectiveness and safety of low-dose versus standard-dose rivaroxaban and apixaban in patients with atrial fibrillation
Source: PLoS One. 2022 Dec 1;17(12):e0277744. doi: 10.1371/journal.pone.0277744 (PMC9714756; doi:10.1371/journal.pone.0277744)

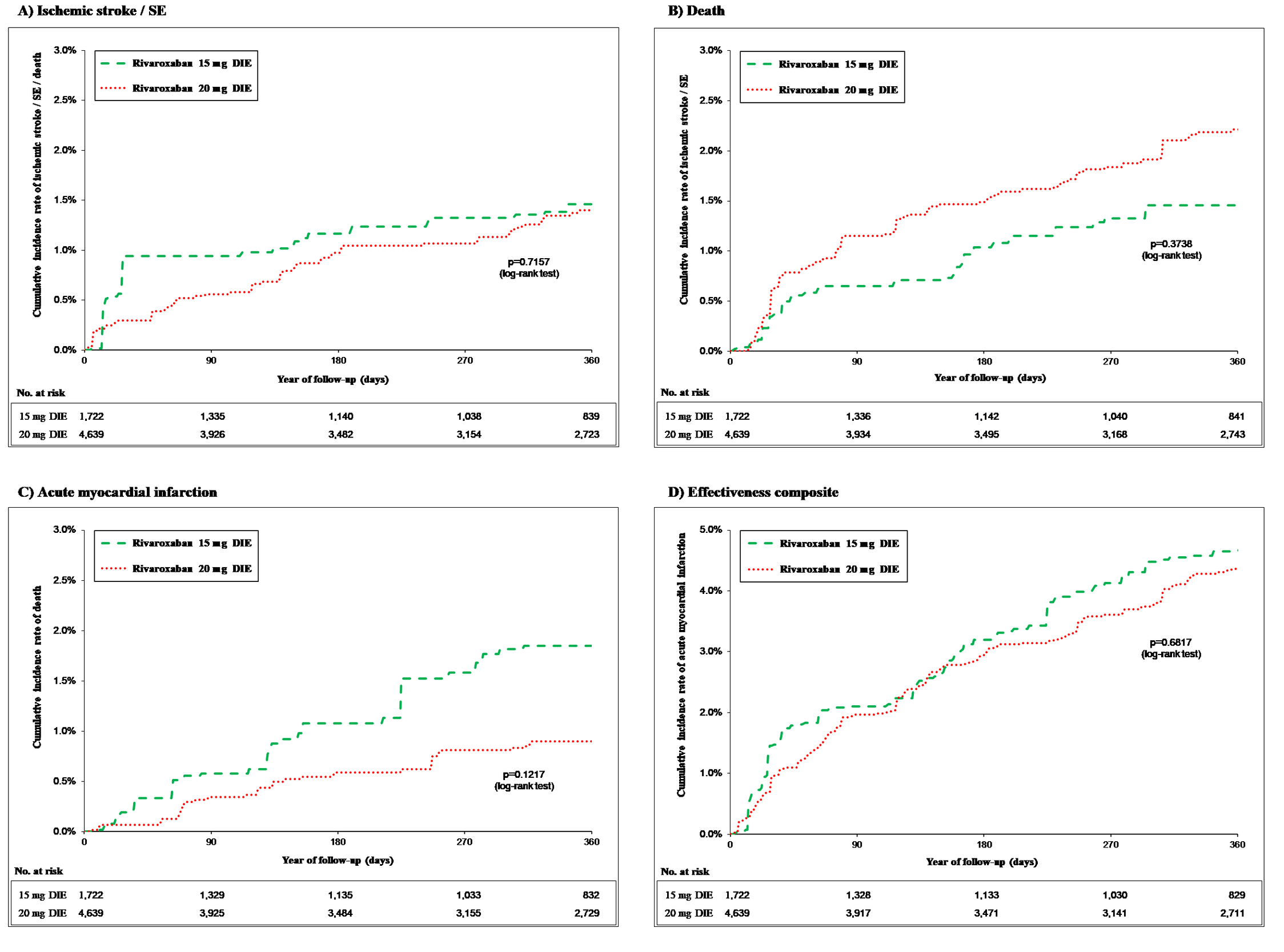

Supplement: S1 Fig — (TIF) [file pone.0277744.s001.tif]

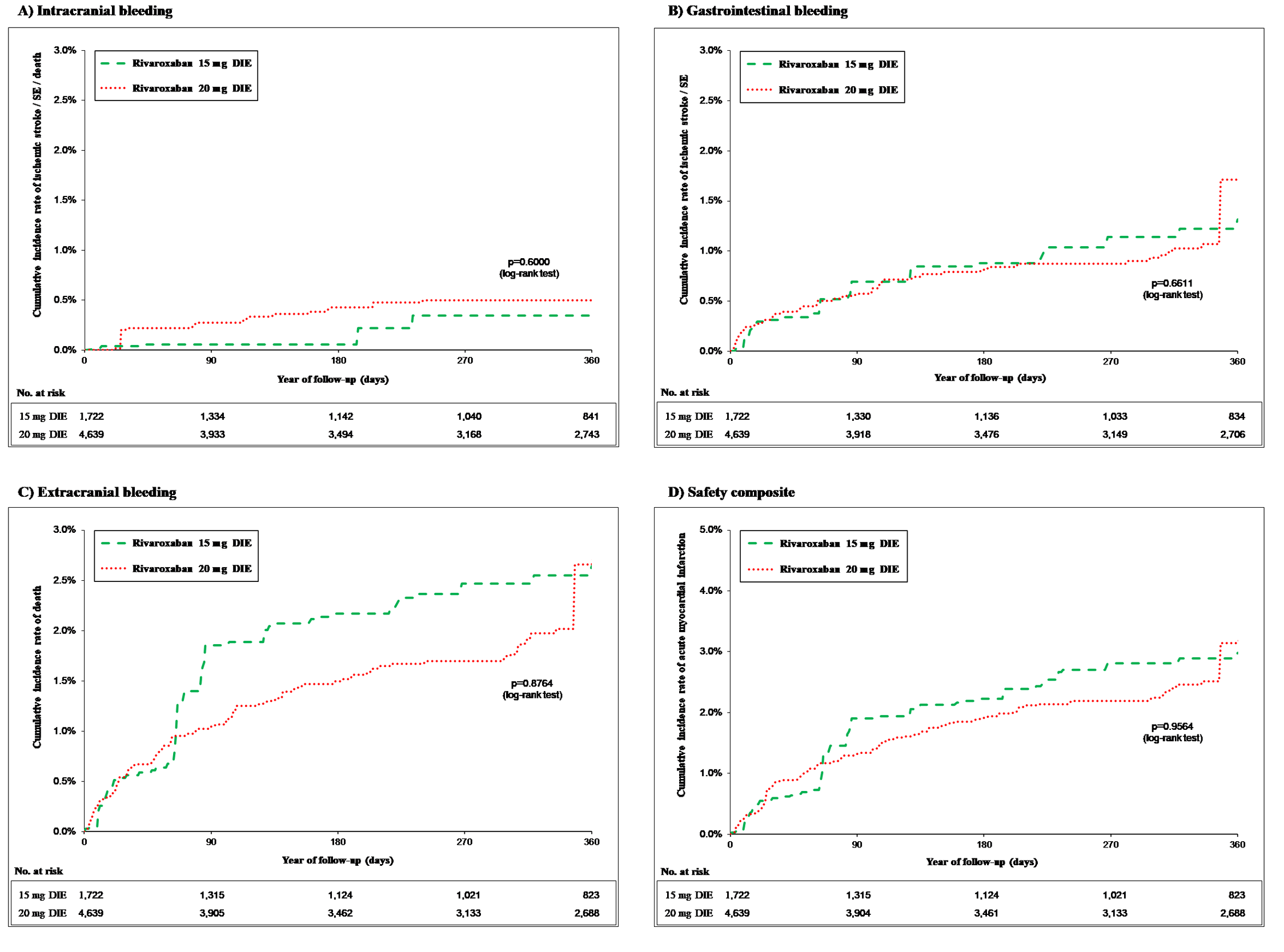

Supplement: S2 Fig — (TIF) [file pone.0277744.s002.tif]

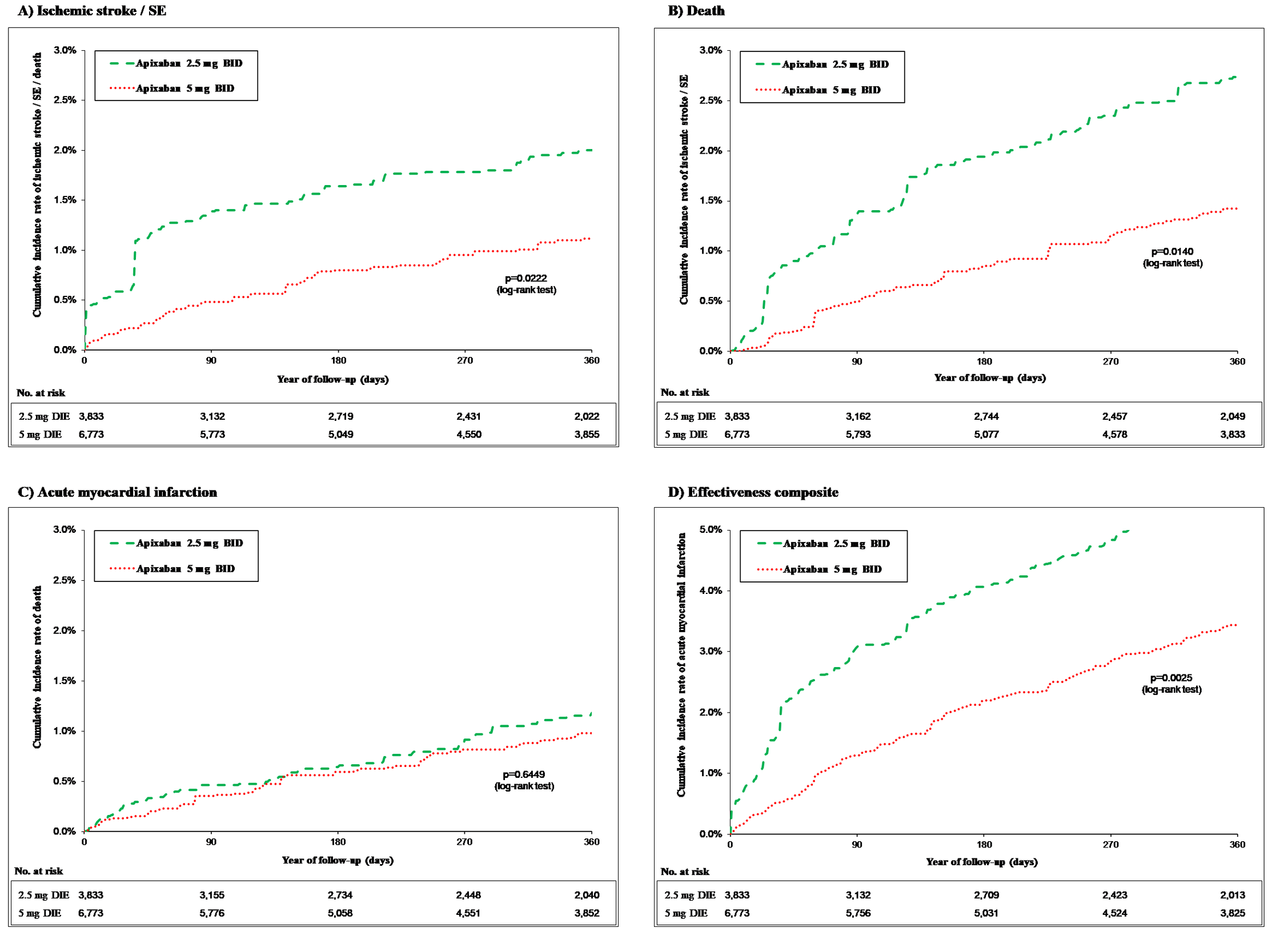

Supplement: S3 Fig — (TIF) [file pone.0277744.s003.tif]

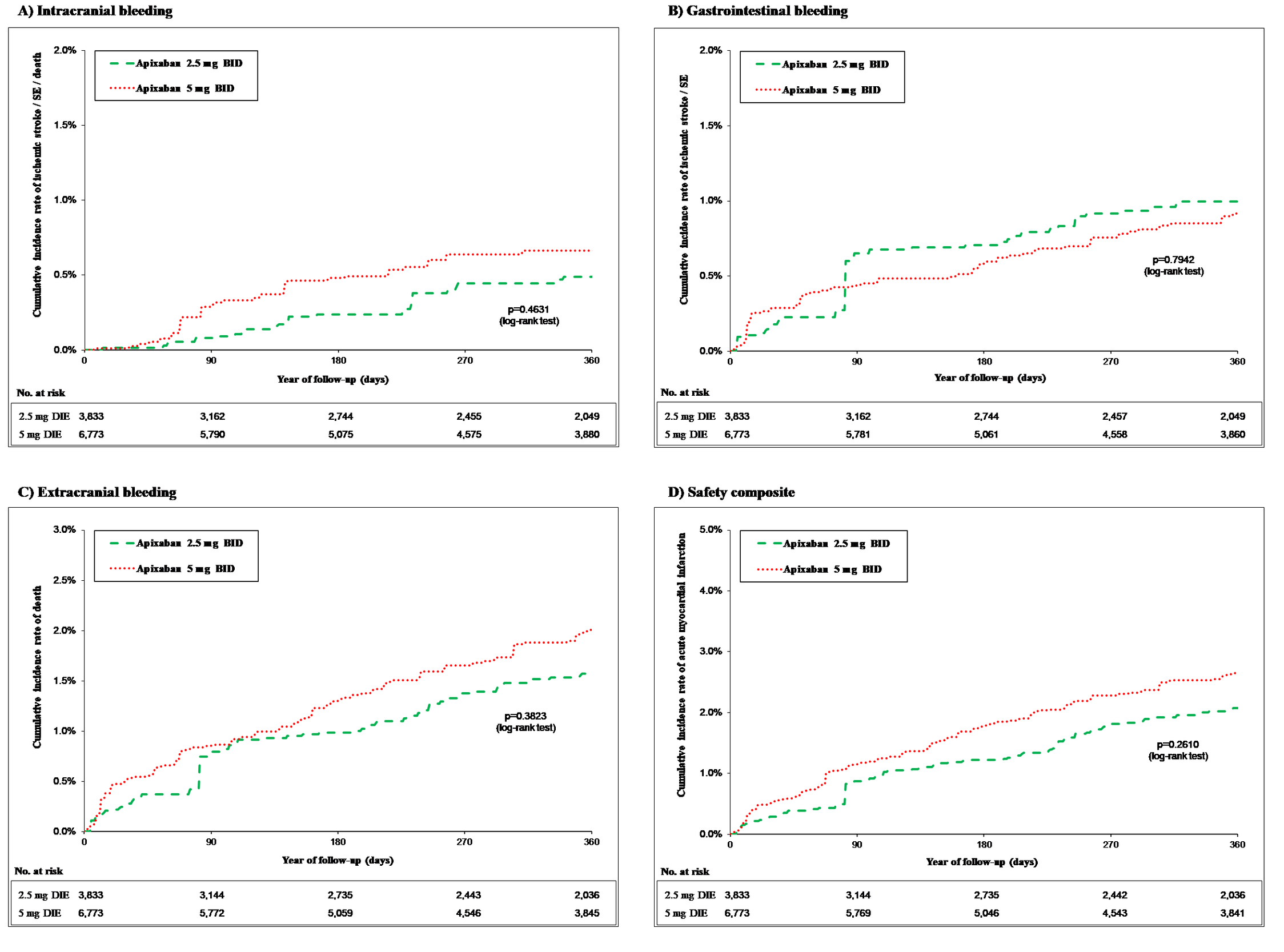

Supplement: S4 Fig — (TIF) [file pone.0277744.s004.tif]
