# Supplementary material for: Effectiveness and safety of low-dose versus standard-dose rivaroxaban and apixaban in patients with atrial fibrillation
Source: PLoS One. 2022 Dec 1;17(12):e0277744. doi: 10.1371/journal.pone.0277744 (PMC9714756; doi:10.1371/journal.pone.0277744)
Supplement: S1 Table — (DOCX) [file pone.0277744.s005.docx]

**S1 Table. Description of data sources.**

The cohort is coming from the Med-Echo administrative databases, which store data on hospital discharges, medical services, and public drug plans, managed by the Régie de l’Assurance Maladie du Québec (RAMQ) [1-4]. The databases were linked using encrypted health insurance numbers. The information from these databases provide a comprehensive picture of the status of hospital admissions.

Data were collected from the “Régie de l’Assurance Maladie du Québec” (RAMQ) and Med-Echo databases, which administer public healthcare insurance programs in the province of Québec, Canada. The Med-Echo database gathers information on acute care hospitalizations, such as date of admission, length of stay, primary and up to 15 secondary diagnoses. The RAMQ data were extracted from three databases. The beneficiary database lists age, gender, socioeconomic status, and date of death. The medical services file contains claims for all inpatient and ambulatory services and diagnostic codes are classified according to the International Classification of Diseases, 9^th^ revision (ICD-10). All surgical procedures codes follow the Canadian classification of diagnostic, therapeutic and surgical procedures [5]. The pharmaceutical database provides data on delivered medication in community pharmacies such as the date of filling, name of the drug, dose, quantity, dosage form, and duration of therapy. The RAMQ covers all Quebec residents for the cost of physician visits, hospitalizations and procedures, and 94% of Quebec citizens aged 65 and older for the drug plan [3, 6].

1. Tamblyn R, Lavoie G, Petrella L, Monette J. The use of prescription claims databases in pharmacoepidemiological research: the accuracy and comprehensiveness of the prescription claims database in Quebec. J Clin Epidemiol. 1995;48(8):999-1009.
2. Eguale T, Winslade N, Hanley JA, Buckeridge DL, Tamblyn R. Enhancing pharmacosurveillance with systematic collection of treatment indication in electronic prescribing: a validation study in Canada. Drug Saf. 2010;33(7):559-67.
3. Wilchesky M, Tamblyn RM, Huang A. Validation of diagnostic codes within medical services claims. J Clin Epidemiol. 2004;57(2):131-41.
4. Tamblyn R, Reid T, Mayo N, McLeod P, Churchill-Smith M. Using medical services claims to assess injuries in the elderly: sensitivity of diagnostic and procedure codes for injury ascertainment. J Clin Epidemiol. 2000;53(2):183-94.
5. Régie de l'assurance maladie du Québec. Rapport annuel de gestion 2005-2006. Quebec: Régie de l'assurance maladie du Québec; 2006.
6. Tamblyn R, Lavoie G, Petrella L, Monette J. The use of prescription claims databases in pharmacoepidemiological research: the accuracy and comprehensiveness of the prescription claims database in Quebec. *J Clin Epidemiol.* Aug 1995;48(8):999-1009.
