# Supplementary material for: Effectiveness and safety of low-dose versus standard-dose rivaroxaban and apixaban in patients with atrial fibrillation
Source: PLoS One. 2022 Dec 1;17(12):e0277744. doi: 10.1371/journal.pone.0277744 (PMC9714756; doi:10.1371/journal.pone.0277744)
Supplement: S3 Table — (DOCX) [file pone.0277744.s007.docx]

**S3 Table. Risk score definition of CHA_2_DS_2_-VASc and modified HAS-BLED.**

| **Risk score definition** | Points if present |
| --- | --- |
|  |  |
| **CHA_2_DS_2_-VASc** |  |
| Congestive heart failure or left ventricular dysfunction | 1 |
| Hypertension | 1 |
| Age 65 – 74 years | 1 |
| Age ≥ 75 years | 2 |
| Diabetes Mellitus | 1 |
| Stroke (ischemic stroke, transient ischemic disease or systemic embolism | 2 |
| Vascular disease (myocardial infarction, peripheral arterial disease or aortic plaque | 1 |
| Sex category (female) | 1 |
|  |  |
| **HAS-BLED** |  |
| Hypertension | 1 |
| Abnormal renal function | 1 |
| Abnormal hepatic function |  |
| Abnormal Stroke (ischemic stroke, transient ischemic disease | 1 |
| Bleeding | 1 |
| Older than > 65 years | 1 |
| Labile international normalized ratio (not available) | 1 |
| Drugs (acetyl salicylic acid, clopidogrel, prasugrel, ticagrelor, ticlopidine, or nonsteroidal anti-inflammatory drugs) in the 1 month preceding the intracranial hemorrhage hospitalization or 1 month after discharge | 1 |
| Alcohol intake | 1 |
