# Supplementary material for: Effectiveness and safety of low-dose versus standard-dose rivaroxaban and apixaban in patients with atrial fibrillation
Source: PLoS One. 2022 Dec 1;17(12):e0277744. doi: 10.1371/journal.pone.0277744 (PMC9714756; doi:10.1371/journal.pone.0277744)
Supplement: S6 Table — (DOCX) [file pone.0277744.s010.docx]

**S6 Table. Medication with pharmacokinetic drug interactions with anticoagulants after inverse probability of treatment weighting.**

| **Medications in the 2 weeks prior the cohort entry - %** | | | | |
| --- | --- | --- | --- | --- |
|  | **Low-dose**  **Rivaroxaban**  **(n=1,722)** | **Standard-dose**  **Rivaroxaban**  **(n=4,639)** | **Low-dose**  **Apixaban**  **(n=3,833)** | **Standard-dose**  **Apixaban**  **(n=6,773)** |
| Anticancer drugs (bosutinib, daunorubirin**,** doxorubicin, etoposide, imatinib,   methotrexate, mitoxanthrone, paclitaxel, vinblastine, vincristine) | 0.4 | 0.7 | 1.1 | 0.7 |
| Antidiarrhoeal agents (loperamide) | 1.0 | 0.6 | 0.3 | 0.5 |
| Antibiotics (clarithromycin, erythromycin) | 0.7 | 0.9 | 0.5 | 0.6 |
| Antidepressant (amitriptyline, citalopram, desipramine, doxepine, fluoxetine, fluvoxamine, imipramine, nortriptyline, paroxetine, trimepramine, venlafaxine) | 13.7 | 11.2 | 11.7 | 12.6 |
| Antihistaminics (cetirizine, desloratadine, fexofenadine) | 0.0 | 0.0 | 0.0 | 0.0 |
| Antipsychotics (risperidone) | 3.1 | 1.6 | 2.3 | 2.8 |
| Antiretroviral (amprenavir, cobicistat, indinavir, lopinavir, nelfinavir, ritonavir, saquinavir) | 0.2 | 0.0 | 0.0 | 0.0 |
| Antifungic (fluconazole, itraconazole, ketoconazole, posaconazole, voriconazole) | 0.3 | 0.5 | 0.3 | 0.2 |
| Cardiovascular drugs (dronaderone, digoxin, diltiazem, propafenone) | 29.6 | 32.9 | 30.0 | 30.5 |
| Antiemetics (domperidone, ondansetron) | 1.6 | 2.1 | 2.7 | 1.9 |
| Antiplatelet agents (clopidogrel, ticagrelor) | 3.2 | 3.8 | 4.4 | 4.4 |
| Beta-blocker (atenolol, talinolol) | 2.9 | 2.9 | 2.1 | 3.0 |
| H2 receptor antagonists (cimetidine, ranitidine) | 0.7 | 0.8 | 1.4 | 1.0 |
| Proton pump inhibitors (esomeprazole, lansoprazole, omeprazole, pantoprazole) | 36.8 | 35.0 | 40.8 | 39.4 |
| Others (atorvastatin, carbamazepine, cyclosporine A**,** chlorpromazine, colchicine, fenofibrate, fluphenazine, phenobarbital, phenytoin, progesterone, midazolam, naprosen, reserpine, tamoxifen, trifluoperazine, | 26.2 | 24.5 | 27.6 | 27.3 |
| **Strong dual inhibitors of CYP3A and P-glycoprotein for rivaroxaban** | | | | |
| Ketoconazole, itraconazole, posaconazole, voriconazole, ritonavir, clarithromycin | 0.8 | 0.9 | - | - |
| **Strong dual inducers of CYP3A and P-glycoprotein for rivaroxaban** | | | | |
| Rifampin, carbamazepine, phenytoin | 1.0 | 0.7 | - | - |
| **Strong dual inhibitors of CYP3A4 and P-glycoprotein for apixaban** | | | | |
| Ketoconazole, itraconazole, posaconazole, voriconazole, ritonavir, clarithromycin | - | - | 0.4 | 0.5 |
| **Strong dual inducers of CYP3A4 and P-glycoprotein for apixaban** | | | | |
| Rifampin, carbamazepine, phenytoin, phenobarbital | - | - | 0.6 | 0.5 |

Foerster KI, Hermann S, Mikus G, Haefeli WE. Drug-drug interactions with direct oral anticoagulants Clin Pharmacokinet. 2020;59(8):967‑80.

Herink MC, Zhuo YF, Williams CD, DeLoughery TG. Clinical management of pharmacokinetic drug interactions with direct oral anticoagulants (DOACs) Drugs. 2019;79(15):1625‑34.

O’Brien FE, Dinan TG, Griffin BT, Cryan JF. Interactions between antidepressants and P-glycoprotein at the blood-brain barrier: Clinical significance of in vitro and in vivo findings. Br J Pharmacol. janv 2012;165(2):289‑312.

Spina E et al. Clinically relevant drug interactions between newer antidepressants and oral anticoagulants. Expert Opinion Drugs and Toxicology 2020 DOI. 10.1080/17425255.2020.1700952.
