# Supplementary material for: Effectiveness and safety of low-dose versus standard-dose rivaroxaban and apixaban in patients with atrial fibrillation
Source: PLoS One. 2022 Dec 1;17(12):e0277744. doi: 10.1371/journal.pone.0277744 (PMC9714756; doi:10.1371/journal.pone.0277744)
Supplement: S7 Table — (DOCX) [file pone.0277744.s011.docx]

**S7 Table.** **Initial cohort and cohort after inverse probability of treatment weighting in low-dose rivaroxaban (15 mg) and standard-dose (20 mg) groups.**

|  | **Initial cohort** | | | **Cohort after IPTW** | | |
| --- | --- | --- | --- | --- | --- | --- |
|  | Rivaroxaban  15 mg  (n=1,722) | Rivaroxaban  20 mg  (n=4,639) | Absolute standardized difference | Rivaroxaban  15 mg  (n=1,722) | Rivaroxaban  20 mg  (n=4,639) | Absolute standardized difference |
| **Age**, mean (SD) | 82.9 (7.2) | 73.1 (9.1) | 1.36 | 75.7 (9.7) | 75.9 (9.8) | 0.03 |
| **Female sex,** (%) | 61.3% | 44.7% | 0.34 | 49.5% | 49.8% | 0.01 |
| **CHA2DS2-VAS Score** (including index hospitalization and 3-y prior to cohort entry), mean (SD) | 4.0 (1.3) | 3.0 (1.4) | 0.78 | 3.2 (1.5) | 3.3 (1.5) | 0.05 |
| **HAS-Bled Score** (including index hospitalization and 3-y prior to cohort entry), mean (SD) | 3.2 (1.3) | 2.5 (1.3) | 0.50 | 2.7 (1.3) | 2.7 (1.3) | 0.04 |
| **Charlson Score Index** (including index hospitalization and 3-y prior to cohort entry), mean (SD) | 4.7 (3.5) | 3.6 (3.2) | 0.32 | 4.2 (3.4) | 4.0 (3.4) | 0.06 |
| **Frailty Score**, mean (SD) | 12.5 (6.8) | 8.9 (6.3) | 0.55 | 10.4 (6.7) | 10.0 (6.6) | 0.05 |
| **Comorbidities** (including index hospitalization and 3-y prior to cohort entry),^¥^  (%) | | | | | | |
| Hypertension | 84.0% | 74.1% | 0.25 | 75.8% | 77.3% | 0.03 |
| Dyslipidemia | 50.4% | 49.0% | 0.03 | 46.4% | 49.5% | 0.06 |
| Diabetes | 31.0% | 31.1% | <0.01 | 33.5% | 31.7% | 0.04 |
| Coronary artery disease | 53.8% | 42.4% | 0.23 | 47.2% | 46.4% | 0.02 |
| Acute myocardial infarction | 14.4% | 8.5% | 0.18 | 11.1% | 10.9% | 0.01 |
| Chronic heart failure | 39.3% | 25.6% | 0.29 | 30.0% | 30.3% | 0.01 |
| Cardiomyopathy | 5.6% | 6.4% | 0.03 | 5.3% | 6.1% | 0.03 |
| Other dysrhythmias | 17.9% | 18.2% | 0.01 | 18.6% | 18.0% | 0.01 |
| Valvular disease | 19.3% | 12.8% | 0.18 | 14.0% | 14.9% | 0.03 |
| Prior cerebrovascular disease including TIA | 18.4% | 13.8% | 0.12 | 13.1% | 15.5% | 0.07 |
| Prior ischemic stroke | 17.8% | 13.6% | 0.11 | 12.9% | 15.1% | 0.07 |
| Peripheral artery disease | 21.2% | 15.8% | 0.14 | 18.9% | 17.8% | 0.03 |
| Chronic renal failure | 40.5% | 16.0% | 0.56 | 27.5% | 24.5% | 0.07 |
| Chronic renal failure < 30 mL/min | 3.0% | 0.9% | 0.15 | 1.4% | 2.0% | 0.05 |
| Acute renal failure | 24.9% | 10.9% | 0.37 | 18.1% | 14.8% | 0.09 |
| Chronic obstructive pulmonary disease/asthma | 37.8% | 36.4% | 0.03 | 37.5% | 36.5% | 0.01 |
| Liver disease | 1.9% | 2.1% | 0.01 | 3.1% | 2.1% | 0.06 |
| Systemic embolism | 2.1% | 1.9% | 0.02 | 2.4% | 1.9% | 0.03 |
| Depression | 12.6% | 10.6% | 0.06 | 10.5% | 10.9% | 0.01 |
| Hypothyroidism | 27.2% | 18.2% | 0.22 | 23.3% | 21.5% | 0.04 |
| Neurologic disorder | 27.3% | 20.0% | 0.17 | 22.8% | 22.8% | <0.01 |
| Prior major bleeding | 30.7% | 20.5% | 0.24 | 25.4% | 23.2% | 0.05 |
| **Malign cancer**, (%) | 25.1% | 24.8% | 0.01 | 27.6% | 25.6% | 0.04 |
| **Medical procedures** (3-y prior to cohort entry), (%) | | | | | | |
| Cardiac catheterization | 2.7% | 3.1% | 0.02 | 3.6% | 3.2% | 0.02 |
| Percutaneous coronary intervention - Stent | 2.7% | 1.8% | 0.06 | 2.2% | 2.3% | 0.01 |
| Coronary artery bypass grafting | 0.6% | 0.5% | 0.01 | 0.5% | 0.6% | 0.01 |
| Implantable cardiac device | <0.1% | <0.1% | 0.01 | <0.1% | <0.1% | 0.01 |
| **Medications** (2 weeks prior to cohort entry), (%) | | | | | | |
| Diuretics | 41.5% | 27.9% | 0.29 | 34.3% | 32.8% | 0.03 |
| Loop diuretics | 33.3% | 20.7% | 0.29 | 26.1% | 25.1% | 0.02 |
| B-Blockers | 64.0% | 62.5% | 0.03 | 62.9% | 63.1% | <0.01 |
| Inhibitors of renin-angiotensin system | 37.1% | 33.9% | 0.07 | 33.5% | 35.0% | 0.03 |
| Calcium channel blockers | 38.5% | 33.5% | 0.10 | 33.7% | 35.2% | 0.03 |
| Statin | 42.0% | 40.4% | 0.03 | 40.8% | 41.2% | 0.01 |
| Antidiabetics | 17.4% | 17.5% | <0.01 | 18.5% | 17.7% | 0.02 |
| Antiplatelet excluding low dose ASA | 5.3% | 2.7% | 0.13 | 3.2% | 3.7% | 0.03 |
| Low dose ASA | 26.8% | 20.9% | 0.14 | 24.0% | 23.8% | 0.01 |
| Proton pump inhibitors | 42.4% | 30.9% | 0.24 | 36.8% | 35.0% | 0.04 |
| NSAIDs | 1.1% | 1.7% | 0.06 | 1.0% | 1.5% | 0.04 |
| Amiodarone or propafenone | 9.6% | 8.9% | 0.02 | 8.7% | 9.0% | 0.01 |
| Digoxin | 11.7% | 8.9% | 0.09 | 8.7% | 9.8% | 0.04 |
| **Antidepressant** |  |  |  |  |  |  |
| SSRI: citalopram, escitalopram, fluoxetine, paroxetine,   sertraline | 9.2% | 7.0% | 0.08 | 8.4% | 7.2% | 0.04 |
| PGP inhibitor use | 60.3% | 51.0% | 0.19 | 53.3% | 54.1% | 0.02 |
| Strong dual inhibitors of CYP3A and PGP for rivaroxaban^‡^ | 0.8% | 0.8% | <0.01 | 0.8% | 0.9% | 0.01 |
| Strong dual inducers of CYP3A and PGP for rivaroxaban^¥^ | 0.7% | 0.6% | 0.01 | 1.0% | 0.7% | 0.03 |
| Number of distinct AHFS classes, mean (SD) | 8.8 (4.2) | 7.6 (4.3) | 0.30 | 8.2 (4.8) | 8.0 (4.4) | 0.04 |
| **Health medical service** (1-y prior cohort entry) |  |  |  |  |  |  |
| Number of specialty visits, mean (SD) | 1.2 (2.1) | 1.2 (2.3) | 0.01 | 1.2 (2.1) | 1.2 (2.3) | 0.01 |
| Number of family physician visits, mean (SD) | 1.4 (3.0) | 1.0 (2.3) | 0.15 | 1.1 (2.4) | 1.0 (2.5) | 0.01 |
| Number of emergency visits, mean (SD) | 3.3 (2.5) | 2.8 (2.5) | 0.21 | 2.9 (2.2) | 2.9 (2.6) | 0.01 |
| **Health hospital service** (3-y prior cohort entry) | | | | | | |
| Number of all-cause hospital admission, mean (SD) | 2.2 (1.7) | 2.0 (1.6) | 0.15 | 2.2 (1.8) | 2.1 (1.7) | 0.07 |
| Length of stay, mean (SD) | 9.7 (11.0) | 7.6 (37.9) | 0.08 | 8.1 (9.3) | 8.0 (35.0) | <0.01 |

***** Strong dual inhibitors of CYP3A4 and PGP for apixaban: ketoconazole, itraconazole, ritonavir, and clarithromycin; ^†^strong dual inducers of CYP3A4 and PGP for apixaban: rifampin, carbamazepine, and phenytoin; **^‡^**strong dual inhibitors of CYP3A and PGP for rivaroxaban: ketoconazole and ritonavir; **^¥^**strong dual inducers of CYP3A and PGP for rivaroxaban: rifampin, carbamazepine, and phenytoin

IPTW: inverse probability of treatment weighting, ASA: acetyl salicylic acid, NSAIDs: nonsteroidal anti-inflammatory drugs, TIA: transient ischemic stroke, PGP: P-glycoprotein, SD: standard deviation, SSRIs: Selective serotonin reuptake inhibitors, AHFS: American Hospital Formulary Service
