# Supplementary material for: Effectiveness and safety of low-dose versus standard-dose rivaroxaban and apixaban in patients with atrial fibrillation
Source: PLoS One. 2022 Dec 1;17(12):e0277744. doi: 10.1371/journal.pone.0277744 (PMC9714756; doi:10.1371/journal.pone.0277744)
Supplement: S8 Table — (DOCX) [file pone.0277744.s012.docx]

**S8 Table. Initial** **cohort and cohort after inverse probability of treatment weighting in low-dose apixaban (2.5 mg) and standard-dose (5 mg) groups.**

|  | **Initial cohort** | | | | **Cohort after IPTW** | | | |
| --- | --- | --- | --- | --- | --- | --- | --- | --- |
|  | Apixaban  2.5 mg  (n=3,833) | Apixaban  5.0.mg  (n=6,773) | Absolute standardized difference | Apixaban  2.5 mg  (n=3,833) | | Apixaban  5.0 mg  (n=6,773) | Absolute standardized difference |  |
| **Age**, mean (SD) | 86.4 (6.1) | 76.3 (8.5) | 1.66 | 79.8 (9.3) | | 80.2 (9.4) | 0.05 |  |
| **Female** **sex,** (%) | 68.5% | 49.1% | 0.40 | 55.8% | | 56.1% | 0.01 |  |
| **CHA2DS2-VAS Score** (including index hospitalization and 3-y prior to cohort entry), mean (SD) | 4.3 (1.2) | 3.4 (1.4) | 0.66 | 3.8 (1.3) | | 3.8 (1.4) | 0.01 |  |
| **HAS-Bled Score** including index hospitalization and 3-y prior cohort entry, mean (SD) | 3.3 (1.3) | 2.9 (1.3) | 0.26 | 3.2 (1.3) | | 3.1 (1.3) | 0.06 |  |
| **Charlson Score Index** (including index hospitalization and 3-y prior to cohort entry), mean (SD) | 4.8 (3.3) | 4.4 (3.4) | 0.12 | 4.7 (3.4) | | 4.6 (3.5) | 0.03 |  |
| **Frailty Score**, mean (SD) | 13.6 (6.9) | 10.2 (6.6) | 0.49 | 11.8 (7.0) | | 11.8 (7.3) | 0.01 |  |
| **Comorbidities** (including index hospitalization and 3-year prior to cohort entry), (%) | | | | | | | |  |
| Hypertension | 83.8% | 79.3% | 0.12 | 82.2% | | 80.7 % | 0.04 |  |
| Dyslipidemia | 49.0% | 54.7% | 0.11 | 54.6% | | 52.6% | 0.04 |  |
| Diabetes | 28.8% | 36.0% | 0.15 | 35.9% | | 33.8% | 0.04 |  |
| Coronary artery disease | 53.5% | 45.8% | 0.15 | 49.9% | | 49.2% | 0.01 |  |
| Acute myocardial infarction | 16.5% | 11.3% | 0.15 | 13.7% | | 13.9% | <0.01 |  |
| Chronic heart failure | 42.6% | 31.8% | 0.22 | 38.0% | | 36.8% | 0.02 |  |
| Cardiomyopathy | 5.0% | 6.5% | 0.06 | 6.1% | | 5.6% | 0.02 |  |
| Other dysrhythmias | 18.9% | 18.5% | 0.01 | 18.3% | | 18.7% | 0.01 |  |
| Valvular disease | 21.9% | 15.3% | 0.17 | 18.0% | | 18.0% | <0.01 |  |
| Prior cerebrovascular disease including TIA | 19.4% | 18.9% | 0.01 | 20.3% | | 19.3% | 0.02 |  |
| Prior stroke/TIA | 18.6% | 18.3% | 0.01 | 19.8% | | 18.7% | 0.03 |  |
| Peripheral artery disease | 21.5% | 18.8% | 0.07 | 20.1% | | 19.5% | 0.02 |  |
| Chronic renal failure | 45.2% | 27.9% | 0.37 | 37.2% | | 35.9% | 0.03 |  |
| Chronic renal failure < 30 mL/min | 3.6% | 2.1% | 0.09 | 3.1% | | 2.8% | 0.02 |  |
| Acute renal failure | 28.1% | 18.4% | 0.23 | 25.0% | | 23.1% | 0.04 |  |
| Chronic obstructive pulmonary disease/asthma | 34.3% | 36.0% | 0.04 | 34.2% | | 36.0% | 0.04 |  |
| Liver disease | 1.5% | 2.4% | 0.06 | 2.3% | | 2.0% | 0.02 |  |
| Systemic embolism | 1.9% | 2.0% | <0.01 | 1.9% | | 2.0% | 0.01 |  |
| Depression | 12.3% | 10.9% | 0.04 | 12.0% | | 11.5% | 0.02 |  |
| Hypothyroidism | 28.3% | 19.8% | 0.20 | 23.4% | | 23.6% | <0.01 |  |
| Neurologic disorder | 31.1% | 22.6% | 0.19 | 26.4% | | 26.7% | 0.01 |  |
| Prior major bleeding | 33.9% | 26.5% | 0.16 | 33.0% | | 30.1% | 0.06 |  |
| **Malign cancer** | 25.1% | 27.0% | 0.04 | 26.8% | | 26.3% | 0.01 |  |
| **Medical procedures** (3-y prior to cohort entry), (%) | | | | | | | |  |
| Cardiac catheterization | 2.6% | 3.7% | 0.06 | 3.2% | | 3.6% | 0.02 |  |
| Percutaneous coronary intervention - Stent | 2.2% | 2.2% | <0.01 | 2.5% | | 2.3% | 0.02 |  |
| Coronary artery bypass grafting | 0.2% | 0.8% | 0.09 | 0.2% | | 0.6% | 0.06 |  |
| Implantable cardiac device | 0.0% | 0.0% | **-** | 0.0% | | 0.0% | **-** |  |
| **Medications** (2 weeks prior to cohort entry), (%) | | | | | | | |  |
| Diuretics | 42.6% | 32.8% | 0.20 | 38.5% | | 37.5% | 0.02 |  |
| Loop diuretics | 36.5% | 25.9% | 0.23 | 31.9% | | 30.9% | 0.02 |  |
| B-Blockers | 65.5% | 65.0% | 0.01 | 61.5% | | 64.3% | 0.06 |  |
| Inhibitors of renin-angiotensin system | 35.2% | 36.5% | 0.03 | 36.3% | | 35.8% | 0.01 |  |
| Calcium channel blockers | 39.5% | 34.9% | 0.09 | 36.9% | | 36.8% | <0.01 |  |
| Statin | 41.4% | 45.1% | 0.07 | 42.2% | | 43.4% | 0.02 |  |
| Antidiabetics | 16.7% | 21.9% | 0.13 | 20.1% | | 20.1% | <0.01 |  |
| Antiplatelet excluding low dose ASA | 5.8% | 3.7% | 0.10 | 4.4% | | 4.4% | <0.01 |  |
| Low dose ASA | 26.5% | 21.4% | 0.12 | 24.4% | | 24.2% | <0.01 |  |
| Proton pump inhibitors | 44.1% | 35.9% | 0.17 | 40.8% | | 39.4% | 0.03 |  |
| NSAIDs | 1.1% | 1.2% | <0.01 | 1.3% | | 1.2% | 0.01 |  |
| Amiodarone or propafenone | 10.1% | 10.1% | <0.01 | 9.2% | | 9.8% | 0.02 |  |
| Digoxin | 10.3% | 8.8% | 0.05 | 9.0% | | 8.9% | <0.01 |  |
| **Antidepressant** |  |  |  |  | |  |  |  |
| SSRI: citalopram, escitalopram, fluoxetine, paroxetine,   sertraline | 10.0% | 8.6% | 0.05 | 9.7% | | 8.9% | 0.03 |  |
| PGP inhibitor use | 60.5% | 54.7% | 0.12 | 57.3% | | 57.1% | <0.01 |  |
| Strong dual inhibitors of CYP3A4 and PGP for apixaban* | 0.5% | 0.6% | 0.01 | 0.4% | | 0.5% | 0.01 |  |
| Strong dual inducers of CYP3A4 and PGP for apixaban^†^ | 0.6% | 0.5% | 0.02 | 0.6% | | 0.5% | <0.01 |  |
| Number of distinct AHFS classes, mean (SD) | 9.1 (4.1) | 8.4 (4.3) | 0.21 | 8.7 (4.3) | | 8.7 (4.3) | 0.01 |  |
| **Health medical service** (1-y prior to cohort entry) | | | | | | | |  |
| Number of specialty visits, mean (SD) | 1.3 (2.7) | 1.4 (2.6) | 0.01 | 1.3 (2.6) | | 1.3 (2.6) | 0.01 |  |
| Number of family physician visits, mean (SD) | 1.3 (3.0) | 0.9 (2.3) | 0.14 | 1.1 (2.6) | | 1.2 (3.6) | 0.03 |  |
| Number of emergency visits, mean (SD) | 3.3 (2.6) | 2.9 (2.5) | 0.15 | 3.1 (2.4) | | 3.1 (2.6) | 0.01 |  |
| **Health hospital service** (3-y prior to cohort entry) | | | | | | | |  |
| Number of all-cause hospital admission, mean (SD) | 2.2 (1.6) | 2.1 (2.1) | 0.02 | 2.3 (1.7) | | 2.2 (2.0) | 0.03 |  |
| Length of stay, mean (SD) | 10.6 (11.7) | 8.3 (10.4) | 0.21 | 9.1 (10.1) | | 9.3 (11.3) | 0.02 |  |

*****Strong dual inhibitors of CYP3A4 and PGP for apixaban: ketoconazole, itraconazole, ritonavir, and clarithromycin; ^†^strong dual inducers of CYP3A4 and PGP for apixaban: rifampin, carbamazepine, and phenytoin; **^‡^**strong dual inhibitors of CYP3A and PGP for rivaroxaban: ketoconazole and ritonavir; **^¥^**strong dual inducers of CYP3A and PGP for rivaroxaban: rifampin, carbamazepine, and phenytoin

IPTW: inverse probability of treatment weighting, ASA: acetyl salicylic acid, NSAIDs: nonsteroidal anti-inflammatory drugs, TIA: transient ischemic stroke, PGP: P-glycoprotein, SD: standard deviation, SSRIs: Selective serotonin reuptake inhibitors, AHFS: American Hospital Formulary Service
