# Supplementary material for: Effectiveness and safety of low-dose versus standard-dose rivaroxaban and apixaban in patients with atrial fibrillation
Source: PLoS One. 2022 Dec 1;17(12):e0277744. doi: 10.1371/journal.pone.0277744 (PMC9714756; doi:10.1371/journal.pone.0277744)
Supplement: S9 Table — (DOCX) [file pone.0277744.s013.docx]

**S9 Table.** **Effectiveness and safety outcomes in the under treatment cohort after inverse probability treatment weighting.**

|  | Rivaroxaban | Rivaroxaban | Apixaban | Apixaban |
| --- | --- | --- | --- | --- |
|  | Low-dose  15 mg | Standard-dose  20 mg | Low-dose  2.5 mg | Standard-dose  5.0 mg |
|  | (n=1,722) | (n=4,639) | (n=3,833) | (n=6,773) |
|  |  |  |  |  |
| **Effectiveness** |  |  |  |  |
|  |  |  |  |  |
| **Stroke (Ischemic only)/SE** |  |  |  |  |
| Events | 21.0 | 53.2 | 65.7 | 62.2 |
| Time to event (days) – mean; median | 139 ; 145 | 149 ; 141 | 113 ; 81 | 115 ; 83 |
| Person-time (year) | 1,181 | 3,548 | 2,781 | 5,168 |
| Event rate per 100 person- years (95%CI) | 1.8 (1.0-2.5) | 1.5 (1.1-1.9) | 2.4 (1.8-2.9) | 1.2 (0.9-1.5) |
| HR (95% CI) | 1.16 (0.70-1.93) p-value: 0.5604 | | 1.95 (1.38-2.76) p-value: 0.0002 | |
|  |  | |  | |
| **All-cause mortality** |  |  |  |  |
| Events | 20.0 | 86.5 | 85.2 | 79.0 |
| Time to event (days) – mean; median | 121 ; 91 | 120 ; 78 | 114 ; 84 | 151 ; 112 |
| Person-time (year) | 1,183 | 3,560 | 2,804 | 5,190 |
| Event rate per 100 person- years (95%CI) | 1.7 (1.0-2.4) | 2.4 (1.9-2.9) | 3.0 (2.4-3.7) | 1.5 (1.2-1.9) |
| HR (95% CI) | 0.68 (0.42-1.11) p-value: 0.1246 | | 1.99 (1.46-2.70) p-value: <0.0001 | |
|  |  | |  | |
| **Acute myocardial infarction** |  |  |  |  |
| Events | 22.9 | 33.1 | 34.8 | 52.9 |
| Time to event (days) – mean; median | 139 ; 132 | 135 ; 123 | 135 ; 130 | 148 ; 125 |
| Person-time (year) | 1,176 | 3,551 | 2,796 | 5,170 |
| Event rate per 100 person- years (95%CI) | 1.9 (1.2-2.7) | 0.9 (0.6-1.2) | 1.2 (0.8-1.7) | 1.0 (0.7-1.3) |
| HR (95% CI) | 2.07 (1.21-3.52) p-value: 0.0077 | | 1.21 (0.79-1.86) p-value: 0.3845 | |
|  |  | |  | |
| **Effectiveness composite** |  |  |  |  |
| Events | 63.1 | 168.5 | 179.3 | 191.0 |
| Time to event (days) – mean; median | 133 ; 133 | 133 ; 117 | 119 ; 85 | 139 ; 107 |
| Person-time (year) | 1,174 | 3,539 | 2,773 | 5,149 |
| Event rate per 100 person- years (95%CI) | 5.4 (4.0-6.7) | 4.8 (4.0-5.5) | 6.5 (5.5-7.4) | 3.7 (3.2-4.2) |
| HR (95% CI) | 1.11 (0.83-1.48) p-value: 0.4864 | | 1.74 (1.41-2.13) p-value: <0.0001 | |
|  |  |  |  |  |
| **Safety** |  |  |  |  |
|  |  |  |  |  |
| **Intracranial bleeding** |  |  |  |  |
| Events | 4.4 | 20.1 | 13.6 | 36.6 |
| Time to event (days) – mean; median | 150 ; 194 | 119 ; 117 | 160 ; 142 | 125 ; 98 |
| Person-time (year) | 1,183 | 3,559 | 2,804 | 5,187 |
| Event rate per 100 person- years (95%CI) | 0.4 (0.0-0.7) | 0.6 (0.3-0.8) | 0.5 (0.2-0.7) | 0.7 (0.5-0.9) |
| HR (95% CI) | 0.65 (0.23-1.81) p-value: 0.4066 | | 0.69 (0.37-1.28) p-value: 0.2354 | |
|  |  | |  | |
| **GI bleeding** |  |  |  |  |
| Events | 17.0 | 61.8 | 30.9 | 51.6 |
| Time to event (days) – mean; median | 113 ; 74 | 111 ; 62 | 137 ; 114 | 131 ; 99 |
| Person-time (year) | 1,178 | 3,542 | 2,798 | 5,175 |
| Event rate per 100 person- years (95%CI) | 1.4 (0.8-2.1) | 1.7 (1.3-2.2) | 1.1 (0.7-1.5) | 1.0 (0.7-1.3) |
| HR (95% CI) | 0.83 (0.48-1.42) p-value: 0.4928 | | 1.10 (0.70-1.72) p-value: 0.6735 | |
|  |  | |  | |
| **Other bleeding** |  |  |  |  |
| Events | 18.8 | 41.7 | 17.0 | 62.8 |
| Time to event (days) – mean; median | 76 ; 66 | 118 ; 63 | 157 ; 155 | 136 ; 122 |
| Person-time (year) | 1,173 | 3,545 | 2,800 | 5,177 |
| Event rate per 100 person- years (95%CI) | 1.6 (1.0-2.5) | 1.2 (0.9-1.6) | 0.6 (0.4-0.9) | 1.2 (0.9-1.5) |
| HR (95% CI) | 1.33 (0.77-2.30) p-value: 0.3029 | | 0.50 (0.29-0.85) p-value: 0.0112 | |
|  |  |  |  |  |
| **Major extracranial bleeding** |  |  |  |  |
| Events | 35.8 | 101.9 | 47.9 | 112.3 |
| Time to event (days) – mean; median | 95 ; 66 | 110 ; 62 | 147 ; 144 | 131 ; 112 |
| Person-time (year) | 1,167 | 3,528 | 2,794 | 5,162 |
| Event rate per 100 person- years (95%CI) | 3.1 (2.1-4.1) | 2.9 (2.3-3.4) | 1.7 (1.2-2.2) | 2.2 (1.8-2.6) |
| HR (95% CI) | 1.05 (0.72-1.54) p-value: 0.7955 | | 0.79 (0.56-1.10) p-value: 0.1614 | |
|  |  | |  | |
| **Safety composite** |  |  |  |  |
| Events | 40.3 | 122.0 | 61.5 | 148.9 |
| Time to event (days) – mean; median | 103 ; 69 | 111 ; 77 | 150 ; 144 | 130 ; 109 |
| Person-time (year) | 1,167 | 3,527 | 2,794 | 5,160 |
| Event rate per 100 person- years (95%CI) | 3.4 (2.4-4.5) | 3.5 (2.8-4.1) | 2.2 (1.7-2.8) | 2.9 (2.4-3.3) |
| HR (95% CI) | 0.98 (0.69-1.41) p-value: 0.9289 | | 0.76 (0.56-1.02) p-value: 0.0709 | |

SE: systemic embolism, HR: hazard ratio, CI: confidence interval, GI: gastro-intestinal
