# Supplementary material for: Effectiveness and safety of low-dose versus standard-dose rivaroxaban and apixaban in patients with atrial fibrillation
Source: PLoS One. 2022 Dec 1;17(12):e0277744. doi: 10.1371/journal.pone.0277744 (PMC9714756; doi:10.1371/journal.pone.0277744)
Supplement: S10 Table — (DOCX) [file pone.0277744.s014.docx]

**S10 Table.** **Effectiveness and safety outcomes in the intent-to-treat cohort after inverse probability of treatment weighting.**

|  | Rivaroxaban | Rivaroxaban | Apixaban | Apixaban |
| --- | --- | --- | --- | --- |
|  | Low-dose  15 mg | Standard-dose  20 mg | Low-dose  2.5 mg | Standard-dose  5.0 mg |
|  | (n=1,722) | (n=4,639) | (n=3,833) | (n=6,773) |
| **Effectiveness** |  |  |  |  |
|  |  |  |  |  |
| **Stroke (Ischemic only)/SE** |  |  |  |  |
| Events | 28.8 | 64.5 | 71.1 | 78.6 |
| Time to event (days) – mean; median | 161 ; 154 | 161 ; 148 | 125 ; 86 | 132 ; 105 |
| Person-time (year) | 1,442 | 4,162 | 3,150 | 5,893 |
| Event rate per 100 person- years (95%CI) | 2.0 (1.3-2.7) | 1.5 (1.2-1.9) | 2.3 (1.7-2.8) | 1.3 (1.0-1.6) |
| HR (95% CI) | 1.28 (0.83-1.99) p-value: 0.2660 | | 1.68 (1.22-2.32) p-value: 0.0016 | |
|  |  | |  | |
| **All-cause mortality** |  |  |  |  |
| Events | 95.3 | 279.4 | 360.5 | 415.8 |
| Time to event (days) – mean; median | 134 ; 105 | 151 ; 124 | 134 ; 106 | 158 ; 137 |
| Person-time (year) | 1,452 | 4,177 | 3,176 | 5,918 |
| Event rate per 100 person- years (95%CI) | 6.6 (5.2-7.9) | 6.7 (5.9-7.5) | 11.3 (10.2-12.5) | 7.0 (6.4-7.7) |
| HR (95% CI) | 0.98 (0.78-1.24) p-value: 0.8517 | | 1.61 (1.40-1.85) p-value:< 0.0001 | |
|  |  | |  | |
| **Acute myocardial infarction** |  |  |  |  |
| Events | 25.3 | 61.3 | 37.6 | 74.0 |
| Time to event (days) – mean; median | 140 ; 132 | 153 ; 134 | 142 ; 138 | 158 ; 131 |
| Person-time (year) | 1,442 | 4,156 | 3,166 | 5,891 |
| Event rate per 100 person- years (95%CI) | 1.8 (1.1-2.4) | 1.5 (1.1-1.8) | 1.2 (0.8-1.6)) | 1.3 (1.0-1.5) |
| HR (95% CI) | 1.19 (0.75-1.89) p-value: 0.4627 | | 0.94 (0.64-1.40) p-value: 0.7691 | |
|  |  | |  | |
| **Effectiveness composite** |  |  |  |  |
| Events | 145.4 | 377.6 | 451.9 | 548.3 |
| Time to event (days) – mean; median | 138 ; 119 | 152 ; 126 | 134 ; 108 | 154 ; 135 |
| Person-time (year) | 1,432 | 4,142 | 3,139 | 5,866 |
| Event rate per 100 person- years (95%CI) | 10.2 (8.5-11.8) | 9.1 (8,2-10.0) | 14.4 (13.1-15.7) | 9.3 (8.6-10.1) |
| HR (95% CI) | 1.11 (0.92-1.34) p-value: 0.2859 | | 1.53 (1.35-1.74) p-value: <0.0001 | |
|  |  |  |  |  |
| **Safety** |  |  |  |  |
|  |  |  |  |  |
| **Intracranial bleeding** |  |  |  |  |
| Events | 6.4 | 20.9 | 17.0 | 43.4 |
| Time to event (days) – mean; median | 156 ; 194 | 114 ; 115 | 167 ; 145 | 158 ; 134 |
| Person-time (year) | 1,451 | 4,174 | 3,176 | 5,911 |
| Event rate per 100 person- years (95%CI) | 0.4 (0.1-0.8) | 0.5 (0.3-0.7) | 0.5 (0.3-0.8) | 0.7 (0.5-1.0) |
| HR (95% CI) | 0.88 (0.36-2.13) p-value: 0.7711 | | 0.73 (0.42-1.28) p-value: 0.2703 | |
|  |  | |  | |
| **GI bleeding** |  |  |  |  |
| Events | 19.2 | 69.2 | 34.5 | 58.7 |
| Time to event (days) – mean; median | 120 ; 86 | 123 ; 80 | 138 ; 124 | 130 ; 99 |
| Person-time (year) | 1,441 | 4,151 | 3,161 | 5,896 |
| Event rate per 100 person- years (95%CI) | 1.3 (0.7-1.9) | 1.7 (1.3-2.1) | 1.1 (0.7-1.5) | 1.0 (0.7-1.2) |
| HR (95% CI) | 0.80 (0.48-1.33) p-value: 0.3860 | | 1.08 (0.71-1.65) p-value: 0.7074 | |
|  |  | |  | |
| **Other bleeding** |  |  |  |  |
| Events | 21.0 | 44.2 | 25.3 | 72.3 |
| Time to event (days) – mean; median | 92 ; 71 | 125 ; 79 | 156 ; 155 | 149 ; 135 |
| Person-time (year) | 1,440 | 4,155 | 3,169 | 5,883 |
| Event rate per 100 person- years (95%CI) | 1.5 (0.9-2.2) | 1.1 (0.8-1.4) | 0.8 (0.6-1.1) | 1.2 (1.0-1.5) |
| HR (95% CI) | 1.36 (0.81-2.29) p-value: 0.2433 | | 0.65 (0.41-1.02) p-value: 0.0610 | |
|  |  |  |  |  |
| **Major extracranial bleeding** |  |  |  |  |
| Events | 40.2 | 111.9 | 59.0 | 126.4 |
| Time to event (days) – mean; median | 107 ; 84 | 120 ; 79 | 148 ; 141 | 135 ; 117 |
| Person-time (year) | 1,430 | 4,129 | 3,154 | 5,862 |
| Event rate per 100 person- years (95%CI) | 2.8 (1.9-3.7) | 2.7 (2.2-3.2) | 1.9 (1.4-2.3) | 2.2 (1.8-2.5) |
| HR (95% CI) | 1.04 (0.72-1.49) p-value: 0.8477 | | 0.86 (0.63-1.18) p-value: 0.3490 | |
|  |  | |  | |
| **Safety composite** |  |  |  |  |
| Events | 46.7 | 132.8 | 75.3 | 169.8 |
| Time to event (days) – mean; median | 115 ; 84 | 119 ; 80 | 153 ; 144 | 140 ; 121 |
| Person-time (year) | 1,428 | 4,127 | 3,153 | 5,855 |
| Event rate per 100 person- years (95%CI) | 3.3 (2.3-4.2) | 3.2 (2.7-3.8) | 2.4 (1.8-2.9) | 2.9 (2.5-3.3) |
| HR (95% CI) | 1.01 (0.72-1.41) p-value: 0.9481 | | 0.82 (0.62-1.08) p-value: 0.1504 | |

SE: systemic embolism, HR: hazard ratio, CI: confidence interval, GI: gastro-intestinal
