# Supplementary material for: Effectiveness and safety of low-dose versus standard-dose rivaroxaban and apixaban in patients with atrial fibrillation
Source: PLoS One. 2022 Dec 1;17(12):e0277744. doi: 10.1371/journal.pone.0277744 (PMC9714756; doi:10.1371/journal.pone.0277744)
Supplement: S11 Table — (DOCX) [file pone.0277744.s015.docx]

**S11 Table. Sensitivity analysis of negative controls after inverse probability of treatment weighting.**

|  | Incident rate of low-dose rivaroxaban  100 PY  (95% CI) | Incident rate of high-dose rivaroxaban  100 PY  (95% CI) | HRs  (95% CI) | P-Value | Incident rate of low-dose apixaban  100 PY  (95% CI) |  | Incident rate  of high-dose apixaban  100 PY  (95% CI) | HRs  (95% CI) | P-Value |
| --- | --- | --- | --- | --- | --- | --- | --- | --- | --- |
| **Negative control** |  |  |  |  |  |  |  |  |  |
| Diabetes complications | 0.23 (0.00-0.50) | 0.66 (0.39-0.93) | 0.34 (0.10-1.20) | 0.0933 | 0.88 (0.54-1.23) |  | 0.38 (0.21-0.54) | 2.34 (1.29-4.25) | 0052 |
| Heart failure | 7.9 (6.3-9.5) | 6.5 (5.7-7.4) | 1.18 (0.93-1.51) | 0.1807 | 10.0 (8.8-11.2) |  | 9.9 (9.0-10.8) | 1.00 (0.86-1.16) | 0.9715 |

CI: confidence interval, HRs: hazard ratios, PY: person-years

For the negative control, we assessed the risk of diabetic complications (ICD-9: 250.1–250.9, 357.2, and 366.41; ICD-10: E10–E14 excluding E10.9, E11.9, E12.9, E13.0, and E14.9) and heart failure (ICD-9: 428; ICD-10: I50) as primary codes of hospitalization, since it should be similar for low-dose and standard-dose DOACs.
