# Supplementary material for: Effectiveness and safety of low-dose versus standard-dose rivaroxaban and apixaban in patients with atrial fibrillation
Source: PLoS One. 2022 Dec 1;17(12):e0277744. doi: 10.1371/journal.pone.0277744 (PMC9714756; doi:10.1371/journal.pone.0277744)
Supplement: S12 Table — (DOCX) [file pone.0277744.s016.docx]

**S12 Table.** **E-values for significant comparisons between low-dose and standard-dose rivaroxaban and apixaban groups under treatment after inverse probability of treatment weighting.**

|  | Hazard ratio (95% CI) | E-value corresponding to the CI bound closest to 1 | E-value for HR point estimate* |
| --- | --- | --- | --- |
| **Low-dose vs Standard-dose rivaroxaban on UT** |  |  |  |
| Acute myocardial infarction | 2.07 (1.21-3.52) | 1.71 | 3.56 |
| **Low-dose vs Standard-dose apixaban on UT** |  |  |  |
| Stroke (Ischemic only)/SE | 1.95 (1.38-2.44) | 2.43 | 3.27 |
| All-cause mortality | 1.99 (1.46-2.70) | 2.28 | 3.39 |
| Effectiveness composite | 1.74 (1.41-2.13) | 2.17 | 2.87 |

SE: systemic embolism, CI: confidence interval, HR: hazard ratio, UT: under treatment
