# Supplementary material for: Effectiveness and safety of low-dose versus standard-dose rivaroxaban and apixaban in patients with atrial fibrillation
Source: PLoS One. 2022 Dec 1;17(12):e0277744. doi: 10.1371/journal.pone.0277744 (PMC9714756; doi:10.1371/journal.pone.0277744)
Supplement: S13 Table — (DOCX) [file pone.0277744.s017.docx]

**S13 Table.** **Distribution of follow-up time and level of adherence at 1-year follow-up.**

|  | Distribution of follow-up time (days) | | Level of adherence (%) | |
| --- | --- | --- | --- | --- |
|  | Mean (95% CI) | Median (Q_1_-Q_3_) | Mean (95% CI) | Median (Q_1_-Q_3_) |
| **As-treated** |  |  |  |  |
| Rivaroxaban 15 mg | 255 (248-261) | 359 (116-365) | 69.7 (67.9-71.4) | 98.4 (31.8-100.0) |
| Rivaroxaban 20 mg | 280 (277-284) | 365 (183-365) | 76.7 (75.7-77.7) | 100.0 (50.1-100.0) |
| Apixaban 2.5 mg | 260 (256-265) | 360 (131-366) | 71.2 (70.1-72.4) | 98.6 (35.9-100.0) |
| Apixaban 5.0 mg | 286 (283-289) | 365 (205-365) | 78.1 (77.3-78.9) | 100.0 (56.2-100.0) |
| **As Intent-to-treat** |  |  |  |  |
| Rivaroxaban 15 mg | 297 (292-303) | 365 (260-365) | 62.5 (60.7-64.3) | 76.4 (21.6-100.0) |
| Rivaroxaban 20 mg | 329 (327-332) | 365 (365-365) | 67.3 (66.2-68.3) | 86.8 (30.7-100.0) |
| Apixaban 2.5 mg | 290 (287-294) | 365 (230-365) | 63.1 (61.9-64.3) | 75.6 (25.2-100.0) |
| Apixaban 5.0 mg | 325 (323-327) | 365 (365-365) | 66.0 (65.2-66.9) | 80.8 (31.8-100.0) |

CI: confidence interval
