# Supplementary material for: Effectiveness and safety of low-dose versus standard-dose rivaroxaban and apixaban in patients with atrial fibrillation
Source: PLoS One. 2022 Dec 1;17(12):e0277744. doi: 10.1371/journal.pone.0277744 (PMC9714756; doi:10.1371/journal.pone.0277744)
Supplement: S14 Table — (DOCX) [file pone.0277744.s018.docx]

**S14 Table. Sample size estimation.**

| Hypothesis HR= 0.90 |  |
| --- | --- |
| Rate of events per 100 person-years | Power 80% |
| 1.0 | 28227 |
| 2.0 | 14114 |
| 3.0 | 9409 |
| 4.0 | 7057 |
| 5.0 | 5646 |
| 6.0 | 4705 |
| Hypothesis HR= 0.85 |  |
| Rate of events per 100 person-years | Power 80% |
| 1.0 | 11864 |
| 2.0 | 5932 |
| 3.0 | 3998 |
| 4.0 | 2982 |
| 5.0 | 2373 |
| 6.0 | 1967 |
| Hypothesis HR= 0.75 |  |
| Rate of events per 100 person-years | Power 80% |
| 1.0 | 3787 |
| 2.0 | 1888 |
| 3.0 | 1270 |
| 4.0 | 950 |
| 5.0 | 755 |
| 6.0 | 633 |
| Hypothesis HR= 0.65 |  |
| Rate of events per 100 person-years | Power 80% |
| 1.0 | 1689 |
| 2.0 | 845 |
| 3.0 | 563 |
| 4.0 | 423 |
| 5.0 | 338 |
| 6.0 | 282 |
| Hypothesis HR= 1.10 |  |
| Rate of events per 100 person-years | Power 80% |
| 1.0 | 34494 |
| 2.0 | 17247 |
| 3.0 | 11498 |
| 4.0 | 8624 |
| 5.0 | 6899 |
| 6.0 | 5749 |
| Hypothesis HR= 1.15 |  |
| Rate of events per 100 person-years | Power 80% |
| 1.0 | 16042 |
| 2.0 | 8021 |
| 3.0 | 5348 |
| 4.0 | 4011 |
| 5.0 | 3209 |
| 6.0 | 2674 |
| Hypothesis HR= 1.25 |  |
| Rate of events per 100 person-years | Power 80% |
| 1.0 | 6293 |
| 2.0 | 3147 |
| 3.0 | 2098 |
| 4.0 | 1574 |
| 5.0 | 1259 |
| 6.0 | 1049 |
| Hypothesis HR= 1.50 |  |
| Rate of events per 100 person-years | Power 80% |
| 1.0 | 1906 |
| 2.0 | 953 |
| 3.0 | 636 |
| 4.0 | 477 |
| 5.0 | 382 |
| 6.0 | 318 |
| Hypothesis HR= 1.75 |  |
| Rate of events per 100 person-years | Power 80% |
| 1.0 | 1001 |
| 2.0 | 501 |
| 3.0 | 334 |
| 4.0 | 251 |
| 5.0 | 201 |
| 6.0 | 167 |
| Hypothesis HR= 2.00 |  |
| Rate of events per 100 person-years | Power 80% |
| 1.0 | 653 |
| 2.0 | 327 |
| 3.0 | 218 |
| 4.0 | 164 |
| 5.0 | 131 |
| 6.0 | 109 |

HR: hazard ratio
