# Supplementary material for: Effectiveness and safety of low-dose versus standard-dose rivaroxaban and apixaban in patients with atrial fibrillation
Source: PLoS One. 2022 Dec 1;17(12):e0277744. doi: 10.1371/journal.pone.0277744 (PMC9714756; doi:10.1371/journal.pone.0277744)
Supplement: S15 Table — (DOCX) [file pone.0277744.s019.docx]

**S15 Table.** **Baseline characteristics after propensity score matching (1:1).**

|  | Low-dose  Rivaroxaban  (n= 1,285) | High-dose  Rivaroxaban  (n= 1,285) | Low-dose  Apixaban  (n= 2,393) | High-dose  Apixaban  (n= 2,393) |
| --- | --- | --- | --- | --- |
| **Age**, mean (SD) | 81.0 (7.0) | 80.7 (6.5) | 84.2 (6.1) | 83.7 (5.8) |
| **Female sex (%)** | 58.4% | 58.1% | 62.4% | 60.8% |
| **CHA_2_DS_2_-VASc Score**, mean (SD) | 3.9 (1.3) | 3.8 (1.2) | 4.2 (1.2) | 4.1 (1.2) |
| **HAS-BLED Score**, mean (SD) | 3.0 (1.3) | 3.0 (1.2) | 3.2 (1.3) | 3.2 (1.3) |
| **Charlson Score Index**, mean (SD) | 4.5 (3.5) | 4.4 (3.5) | 4.8 (3.4) | 4.7 (3.5) |
| **Frailty Index**, mean (SD) | 11.6 (6.6) | 11.2 (6.6) | 12.7 (6.7) | 12.3 (6.7) |
| **Comorbidities (including index hospitalization and 3-year prior to cohort entry), (%)** | | | | |
| Hypertension | 82.4 % | 82.0% | 83.1 % | 83.5% |
| Dyslipidemia | 51.0% | 49.2% | 52.3% | 52.3% |
| Diabetes | 31.1% | 31.4% | 32.1% | 32.6% |
| Coronary artery disease | 52.1% | 51.5% | 52.2% | 50.7% |
| Acute myocardial infarction | 14.4% | 12.7% | 15.1% | 14.4% |
| Chronic heart failure | 33.7% | 35.6% | 39.0% | 38.5% |
| Cardiomyopathy | 5.5% | 6.1% | 5.1% | 5.0% |
| Other dysrhythmias | 17.9% | 17.0% | 19.1% | 19.4% |
| Valvular disease | 17.9% | 17.5% | 20.3% | 19.2% |
| Prior cerebrovascular disease including TIA | 17.7% | 16.3% | 19.7% | 19.6% |
| Prior ischemic stroke | 17.2% | 16.0% | 19.1% | 18.8% |
| Peripheral artery disease | 20.9% | 20.5% | 21.2% | 21.0% |
| Chronic renal failure | 35.0% | 32.7% | 39.9% | 38.2% |
| Chronic renal failure < 30 mL/min | 2.7% | 1.9% | 3.5% | 3.4% |
| Acute renal failure | 21.5% | 21.2% | 24.9% | 24.4% |
| Chronic obstructive pulmonary disease/asthma | 37.0% | 36.9% | 35.1% | 34.4% |
| Liver disease | 1.9% | 2.5% | 1.8% | 2.0% |
| Systemic embolism | 2.3% | 2.3% | 2.1% | 1.8% |
| Depression | 11.9% | 11.7% | 11.8% | 11.5% |
| Hypothyroidism | 25.4% | 26.4% | 26.8% | 25.6% |
| Neurologic disorder | 26.1% | 25.6% | 29.4% | 28.3% |
| Prior major bleeding | 27.4% | 28.3% | 32.5% | 32.4% |
| Malignant cancer | 26.2% | 26.4% | 26.7% | 27.6% |
| **Medical procedures (3 years prior to the index claim), (%)** | | | | |
| Cardiac catheterization | 3.2% | 3.3% | 2.8% | 3.0% |
| Percutaneous coronary intervention – Stent | 2.4% | 2.0% | 2.3% | 2.1% |
| Coronary artery bypass grafting | 0.8% | 0.7% | 0.3% | 0.3% |
| Implantable cardiac device | 0.1% | 0.1% | 0.0% | 0.0% |
| **Medications (in the 2-week prior the index claim), (%)** |  |  |  |  |
| Diuretics | 36.9% | 38.1% | 40.0% | 39.8% |
| Loop diuretics | 28.5% | 29.5% | 33.4% | 33.1% |
| B-Blockers | 62.9% | 63.4% | 65.8% | 65.0% |
| Inhibitors of renin-angiotensin system | 36.7% | 36.9% | 35.9% | 36.4% |
| Calcium channel blockers | 37.8% | 37.3% | 38.3% | 37.8% |
| Statin | 42.5% | 42.4% | 43.5% | 43.5% |
| Antidiabetics | 17.3% | 18.0% | 18.9% | 18.9% |
| Antiplatelet excluding low dose ASA | 4.5% | 3.9% | 5.2% | 4.5% |
| Low dose ASA | 25.6% | 25.2% | 24.9% | 25.0% |
| Proton pump inhibitors | 39.1% | 39.6% | 41.5% | 41.8% |
| NSAIDs | 1.2% | 1.1% | 1.2% | 1.3% |
| Amiodarone or propafenone | 9.2% | 10.3% | 9.6% | 10.0% |
| Digoxin | 10.1% | 10.6% | 9.5% | 9.7% |
| **Antidepressant** |  |  |  |  |
| SSRI (citalopram, escitalopram, fluoxetine, paroxetine, sertraline) | 9.0% | 8.3% | 9.6% | 10.1% |
| PGP inhibitor use | 57.6% | 58.3% | 57.8% | 58.6% |
| Strong dual inhibitors of CYP3A and PGP for rivaroxaban^‡^ | 0.9% | 1.0% | - | - |
| Strong dual inducers of CYP3A and PGP for rivaroxaban^¥^ | 0.9% | 0.9% | - | - |
| Strong dual inhibitors of CYP3A4 and PGP for apixaban* | - | - | 0.4% | 0.5% |
| Strong dual inducers of CYP3A4 and PGP for apixaban† | - | - | 0.7% | 0.6% |
| Number of distinct AHFS classes, mean (SD) | 8.5 (4.2) | 8.6 (4.4) | 8.9 (4.1) | 8.9 (4.2) |
| **Health medical service in 1-y prior the index claim** |  |  |  |  |
| Number of specialty visits, mean (SD) | 1.2 (2.7) | 1.3 (3.0) | 1.2 (2.7) | 1.1 (2.7) |
| Number of family physician visits, mean (SD) | 1.3 (2.3) | 1.3 (2.7) | 1.3 (2.4) | 1.4 (3.0) |
| Number of emergency visits, mean (SD) | 3.1 (2.4) | 3.1 (2.7) | 3.1 (2.5) | 3.1 (2.5) |
| **Health hospital service in 3-year prior the index claim** |  |  |  |  |
| Number of all-cause hospital admission, mean (SD) | 2.2 (1.7) | 2.2 (1.7) | 2.2 (1.6) | 2.2 (1.6) |
| Length of stay, mean (SD) | 8.9 (10.4) | 8.5 (9.8) | 9.7 (11.0) | 9.7 (11.3) |

***** Strong dual inhibitors of CYP3A4 and PGP for apixaban: ketoconazole, itraconazole, ritonavir, clarithromycin; ^†:^ Strong dual inducers of CYP3A4 and PGP for apixaban: Rifampin, carbamazepine, phenytoin; ^‡^: Strong dual inhibitors of CYP3A and PGP for rivaroxaban: ketoconazole, ritonavir; ^¥^: Strong dual inducers of CYP3A and PGP for rivaroxaban: rifampin, carbamazepine, phenytoin.

ASA: acetyl salicylic acid, NSAIDs: Nonsteroidal anti-inflammatory drugs, SD: standard deviation, TIA: transient ischemic stroke, PGP: P-glycoprotein, SSRI: selective serotonin reuptake inhibitors; AHFS: American Hospital Formulary Service
