# Supplementary material for: Effectiveness and safety of low-dose versus standard-dose rivaroxaban and apixaban in patients with atrial fibrillation
Source: PLoS One. 2022 Dec 1;17(12):e0277744. doi: 10.1371/journal.pone.0277744 (PMC9714756; doi:10.1371/journal.pone.0277744)
Supplement: S16 Table — (DOCX) [file pone.0277744.s020.docx]

**S16 Table.** **Comparative effectiveness and safety outcomes in propensity-score-matched cohorts on-treatment (UT) and intent-to-treat (ITT).**

|  |  | Rivaroxaban | | Rivaroxaban | HR  (95% CI) | P-value | Apixaban | Apixaban | HR  (95% CI) | P-value |
| --- | --- | --- | --- | --- | --- | --- | --- | --- | --- | --- |
|  |  | Low-dose | | Standard-dose |  |  | Low-dose | Standard-dose |  |  |
|  |  | (n=1,285) | | (n=1,285) |  |  | (n=2,393) | (n=2,393) |  |  |
| **Effectiveness** |  |  | |  |  |  |  |  |  |  |
|  |  |  | |  |  |  |  |  |  |  |
| **Stroke (Ischemic only)/SE** |  |  |  | |  |  |  |  |  |  |
| Event rate per 100 person-years 95%CI * | UT | 1.6 (0.7-2.4) | 1.1 (0.4-1.8) | | 1.37 (0.60-3.13) | 0.4514 | 2.1 (1.4-2.8) | 1.4 (0.8-2.0) | 1.50 (0.88-2.54) | 0.1366 |
| Event rate per 100 person-years 95%CI * | ITT | 1.9 (1.0-2.7) | 1.4 (0.7-2.1) | | 1.29 (0.67-2.48) | 0.4527 | 2.2 (1.6-2.9) | 1.5 (1.0-2.1) | 1.43 (0.90-2.27) | 0.1273 |
|  |  |  |  | |  |  |  |  |  |  |
| **All-cause mortality** |  |  | | |  |  |  | |  |  |
| Event rate per 100 person-years 95%CI * | UT | 2.7 (1.5-3.8) | 2.1 (1.2-3.1) | | 1.22 (0.66-2.26) | 0.5236 | 3.4 (2.5-4.3) | 1.4 (0.9-2.0) | 2.36 (1.46-3.80) | 0.0004 |
| Event rate per 100 person-years 95%CI * | ITT | 9.6 (7.8-11.5) | 7.2 (5.6-8.7) | | 1.34 (0.99-2.79) | 0.0513 | 12.0 (10.5-13.6) | 7.0 (5.8-8.1) | 1.71 (1.39-2.10) | <0.0001 |
|  |  |  |  | |  |  |  |  |  |  |
| **Acute myocardial infarction** |  |  |  | |  |  |  |  |  |  |
| Event rate per 100 person-years 95%CI * | UT | 2.5 (1.5-3.6) | 1.4 (0.6-2.1) | | 1.86 (0.92-3.78) | 0.0860 | 1.5 (0.9-2.1) | 1.4 (0.8-2.0) | 1.10 (0.62-1.93) | 0.7487 |
| Event rate per 100 person-years 95%CI * | ITT | 2.3 (1.4-3.3) | 1.6 (0.9-2.4) | | 1.43 (0.78-2.62) | 0.2464 | 1.5 (1.0-2.1) | 1.4 (0.9-2.0) | 1.07 (0.64-1.78) | 0.7970 |
|  |  |  |  | |  |  |  |  |  |  |
| **Effectiveness composite** |  |  |  | |  |  |  |  |  |  |
| Event rate per 100 person-years 95%CI * | UT | 6.6 (4.8-8.3) | 4.4 (3.0-5.8) | | 1.47 (0.97-2.22) | 0.0672 | 6.7 (5.5-8.0) | 4.2 (3.2-5.2) | 1.61 (1.19-2.17) | 0.0020 |
| Event rate per 100 person-years 95%CI * | ITT | 13.4 (11.2-15.6) | 9.7 (7.8-11.5) | | 1.37 (1.07-1.76) | 0.0140 | 15.2 (13.5-17.0) | 9.8 (8.4-11.1) | 1.55 (1.30-1.86) | <0.0001 |
|  |  |  |  | |  |  |  |  |  |  |
| **Safety** |  |  |  | |  |  |  |  |  |  |
|  |  |  |  | |  |  |  |  |  |  |
| **Intracranial bleeding** |  |  |  | |  |  |  |  |  |  |
| Event rate per 100 person-years 95%CI * | UT | 0.4 (0.0-0.8) | 0.3 (0.0-0.7) | | 1.07 (0.22-5.29) | 0.9360 | 0.7 (0.3-1.1) | 0.8 (0.4-1.2) | 0.86 (0.38-1.91) | 0.7037 |
| Event rate per 100 person-years 95%CI * | ITT | 0.5 (0.1-0.9) | 0.3 (0.0-0.6) | | 1.73 (0.41-7.22) | 0.4553 | 0.8 (0.4-1.2) | 0.8 (0.4-1.2) | 1.04 (0.52-2.08) | 0.9140 |
|  |  |  |  | |  |  |  |  |  |  |
| **GI bleeding** |  |  |  | |  |  |  |  |  |  |
| Event rate per 100 person-years 95%CI * | UT | 1.8 (0.9-2.7) | 2.2 (1.2-3.1) | | 0.83 (0.42-1.63) | 0.5832 | 0.7 (0.3-1.1) | 1.0 (0.5-1.5) | 0.71 (0.34-1.49) | 0.3679 |
| Event rate per 100 person-years 95%CI * | ITT | 1.9 (1.1-2.7) | 2.1 (1.2-2.9) | | 0.89 (0.49-1.62) | 0.7032 | 0.9 (0.4-1.4) | 1.1 (0.6-1.5) | 0.84 (0.45-1.57) | 0.5899 |
|  |  |  |  | |  |  |  |  |  |  |
| **Extracranial bleeding** |  |  |  | |  |  |  |  |  |  |
| Event rate per 100 person-years 95%CI * | UT | 3.3 (2.0-4.5) | 3.9 (2.6-5.2) | | 0.84 (0.50-1.38) | 0.4849 | 1.6 (1.0-2.2) | 2.4 (1.7-3.2) | 0.66 (0.40-1.07) | 0.0939 |
| Event rate per 100 person-years 95%CI * | ITT | 3.5 (2.4-4.6) | 3.6 (2.4-4.7) | | 0.97 (0.62-1.52) | 0.8937 | 1.7 (1.1-2.2) | 2.4 (1.7-3.1) | 0.69 (0.44-1.07) | 0.0996 |
| **Safety composite** |  |  |  | |  |  |  |  |  |  |
| Event rate per 100 person-years 95%CI * | UT | 3.6 (2.3-4.9) | 4.2 (2.9-5.6) | | 0.85 (0.53-1.38) | 0.5202 | 2.3 (1.5-3.0) | 3.2 (2.3-4.1) | 0.71 (0.46-1.07) | 0.1026 |
| Event rate per 100 person-years 95%CI * | ITT | 4.0 (2.8-5.2) | 3.8 (2.7-5.0) | | 1.02 (0.67-1.57) | 0.9153 | 2.4 (1.7-3.1) | 3.2 (2.4-4.0) | 0.76 (0.52-1.10) | 0.1477 |

* Crude rate

SE: systemic embolism, CI: confidence interval, HR: hazard ratio, GI: gastro-intestinal
