# Supplementary material for: Effectiveness and safety of low-dose versus standard-dose rivaroxaban and apixaban in patients with atrial fibrillation
Source: PLoS One. 2022 Dec 1;17(12):e0277744. doi: 10.1371/journal.pone.0277744 (PMC9714756; doi:10.1371/journal.pone.0277744)
Supplement: S17 Table — (DOCX) [file pone.0277744.s021.docx]

**S17 Table.** **Effectiveness and safety outcomes in the under treatment cohort from raw data, IPTW method and PS match 1:1.**

|  | Rivaroxaban | Rivaroxaban | Apixaban | Apixaban |
| --- | --- | --- | --- | --- |
|  | Low-dose  15 mg | Standard-dose  20 mg | Low-dose  2.5 mg | Standard-dose  5.0 mg |
| **Sample size** |  |  |  |  |
| Raw data | (n=1,722) | (n=4,639) | (n=3,833) | (n=6,773) |
| IPTW method | (n=1,722) | (n=4,639) | (n=3,833) | (n=6,773) |
| PS-Match method | (n=1,285) | (n=1,285) | (n=2,393) | (n=2,393) |
| **Effectiveness** |  |  |  |  |
| **Stroke (ischemic only/SE)** |  |  |  |  |
| **Raw data** |  |  |  |  |
| Event rate per 100 persons-years (95%CI) | 1.4 (0.7-2.1) | 1.3 (0.9-1.6) | 2.3 (1.7-2.8) | 1.1 (0.8-1.3) |
| Adjusted^*^ HR (95% CI) | 0.88 (0.47-1.66) | | 1.50 (0.97-2.32) | |
| **IPTW method** |  |  |  |  |
| Event rate per 100 persons-years (95%CI) | 1.8 (1.0–2.5) | 1.5 (1.1–1.9) | 2.4 (1.8–2.9) | 1.2 (0.9–1.5) |
| Marginal HR (95% CI) | 1.16 (0.70-1.93) | | 1.95 (1.38-2.76) | |
| **PS-Match method** |  |  |  |  |
| Event rate per 100 persons-years (95%CI) | 1.6 (0.7-2.4) | 1.1 (0.4-1.8) | 2.1 (1.4-2.8) | 1.4 (0.8-2.0) |
| HR (95% CI) | 1.37 (0.60-3.13) | | 1.50 (0.88-2.54) | |
| **All-cause mortality** |  |  |  |  |
| **Raw data** |  |  |  |  |
| Event rate per 100 persons-years (95%CI) | 2.8 (1.9-3.8) | 1.9 (1.5-2.4) | 3.8 (3.1-4.5) | 1.4 (1.0-1.7) |
| Adjusted^*^ HR (95% CI) | 0.81 (0.50-1.30) | | 2.06 (1.43-2.97) | |
| **IPTW method** |  |  |  |  |
| Event rate per 100 persons-years (95%CI) | 1.7 (1.0–2.4) | 2.4 (1.9–2.9) | 3.0 (2.4–3.7) | 1.5 (1.2–1.9) |
| Marginal HR (95% CI) | 0.68 (0.42-1.11) | | 1.99 (1.46-2.70) | |
| **PS-Match method** |  |  |  |  |
| Event rate per 100 persons-years (95%CI) | 2.7 (1.5-3.8) | 2.1 (1.2-3.1) | 3.4 (2.5-4.3) | 1.4 (0.9-2.0) |
| HR (95% CI) | 1.22 (0.66-2.26) | | 2.36 (1.46-3.80) | |
| **Acute myocardial infarction** |  |  |  |  |
| **Raw data** |  |  |  |  |
| Event rate per 100 persons-years (95%CI) | 2.2 (1.3-3.0) | 0.8 (0.5-1.1) | 2.0 (1.5-2.5) | 0.9 (0.6-1.1) |
| Adjusted^*^ HR (95% CI) | 2.10 (1.13-3.92) | | 1.41 (0.88-2.23) | |
| **IPTW method** |  |  |  |  |
| Event rate per 100 persons-years (95%CI) | 1.9 (1.2–2.7) | 0.9 (1.2–2.7) | 1.2 (0.8–1.7) | 1.0 (0.7–1.3) |
| Marginal HR (95% CI) | 2.07 (1.21-3.52) | | 1.21 (0.79-1.86) | |
| **PS-Match method** |  |  |  |  |
| Event rate per 100 persons-years (95%CI) | 2.5 (1.5-3.6) | 1.4 (0.6-2.1) | 1.5 (0.9-2.1) | 1.4 (0.8-2.0) |
| HR (95% CI) | 1.86 (0.92-3.78) | | 1.10 (0.62-1.93) | |
| **Effectiveness composite** |  |  |  |  |
| **Raw data** |  |  |  |  |
| Event rate per 100 persons-years (95%CI) | 6.3 (4.9-7.7) | 3.9 (3.2-4.6) | 7.6 (6.6-8.6) | 3.3 (2.8-3.8) |
| Adjusted^*^ HR (95% CI) | 1.07 (0.77-1.48) | | 1.64 (1.29-2.10) | |
| **IPTW method** |  |  |  |  |
| Event rate per 100 persons-years (95%CI) | 5.4 (4.0–6.7) | 4.8 (4.0–5.5) | 6.5 (5.5–7.4) | 3.7 (3.2–4.2) |
| Marginal HR (95% CI) | 1.11 (0.83-1.48) | | 1.74 (1.41-2.13) | |
| **PS-Match method** |  |  |  |  |
| Event rate per 100 persons-years (95%CI) | 6.6 (4.8-8.3) | 4.4 (3.0-5.8) | 6.7 (5.5-8.0) | 4.2 (3.2-5.2) |
| HR (95% CI) | 1.47 (0.97-2.22) | | 1.61 (1.19-2.17) | |
| **Safety** |  |  |  |  |
| **Intracranial bleeding** |  |  |  |  |
| **Raw data** |  |  |  |  |
| Event rate per 100 persons-years (95%CI) | 0.6 (0.2-1.0) | 0.4 (0.2-0.6) | 0.7 (0.4-1.1) | 0.4 (0.3-0.6) |
| Adjusted^*^ HR (95% CI) | 0.80 (0.28-2.32) | | 0.75 (0.37-1.52) | |
| **IPTW method** |  |  |  |  |
| Event rate per 100 persons-years (95%CI) | 0.4 (0.0–0.7) | 0.6 (0.3–0.8) | 0.5 (0.2–0.7) | 0.7 (0.5–0.9) |
| Marginal HR (95% CI) | 0.65 (0.23-1.81) | | 0.69 (0.37-1.28) | |
| **PS-Match method** |  |  |  |  |
| Event rate per 100 persons-years (95%CI) | 0.4 (0.0-0.8) | 0.3 (0.0-0.7) | 0.7 (0.3-1.1) | 0.8 (0.4-1.2) |
| HR (95% CI) | 1.07 (0.22-5.29) | | 0.86 (0.38-1.91) | |
| **GI bleeding** |  |  |  |  |
| **Raw data** |  |  |  |  |
| Event rate per 100 persons-years (95%CI) | 1.7 (0.9-2.4) | 1.2 (0.8-1.5) | 1.0 (0.6-1.3) | 0.8 (0.6-1.1) |
| Adjusted^*^ HR (95% CI) | 0.84 (0.46-1.56) | | 0.94 (0.53-1.67) | |
| **IPTW method** |  |  |  |  |
| Event rate per 100 persons-years (95%CI) | 1.4 (0.8–2.1) | 1.7 (1.3–2.2) | 1.1 (0.7–1.5) | 1.0 (0.7–1.3) |
| Marginal HR (95% CI) | 0.83 (0.48-1.42) | | 1.10 (0.70-1.72) | |
| **PS-Match method** |  |  |  |  |
| Event rate per 100 persons-years (95%CI) | 1.8 (0.9-2.7) | 2.2 (1.2-3.1) | 0.7 (0.3-1.1) | 1.0 (0.5-1.5) |
| HR (95% CI) | 0.83 (0.42-1.63) | | 0.71 (0.34-1.49) | |
| **Extracranial bleeding** |  |  |  |  |
| **Raw data** |  |  |  |  |
| Event rate per 100 persons-years (95%CI) | 3.3 (2.3-4.3) | 2.2 (1.7-2.7) | 1.8 (1.3-2.3) | 1.6 (1.3-2.0) |
| Adjusted^*^ HR (95% CI) | 0.85 (0.55-1.33) | | 0.77 (0.51-1.17) | |
| **IPTW method** |  |  |  |  |
| Event rate per 100 persons-years (95%CI) | 3.1 (2.1–4.1) | 2.9 (2.3–3.4) | 1.7 (1.2–2.2) | 2.2 (1.8–2.6) |
| Marginal HR (95% CI) | 1.05 (0.72-1.54) | | 0.79 (0.56-1.10) | |
| **PS-Match method** |  |  |  |  |
| Event rate per 100 persons-years (95%CI) | 3.3 (2.0-4.5) | 3.9 (2.6-5.2) | 1.6 (1.0-2.2) | 2.4 (1.7-3.2) |
| HR (95% CI) | 0.84 (0.50-1.38) | | 0.66 (0.40-1.07) | |
| **Safety composite** |  |  |  |  |
| **Raw data** |  |  |  |  |
| Event rate per 100 persons-years (95%CI) | 3.9 (2.8-5.0) | 2.6 (2.0-3.1) | 2.5 (1.9-3.1) | 2.1 (1.7-2.4) |
| Adjusted^*^ HR (95% CI) | 0.85 (0.57-1.27) | | 0.77 (0.54-1.10) | |
| **IPTW method** |  |  |  |  |
| Event rate per 100 persons-years (95%CI) | 3.4 (2.4–4.5) | 3.5 (2.8–4.1) | 2.2 (1.7–2.8) | 2.9 (2.4–3.3) |
| Marginal HR (95% CI) | 0.98 (0.69-1.41) | | 0.76 (0.56-1.02) | |
| **PS-Match method** |  |  |  |  |
| Event rate per 100 persons-years (95%CI) | 3.6 (2.3-4.9) | 4.2 (2.9-5.6) | 2.3 (1.5-3.0) | 3.2 (2.3-4.1) |
| HR (95% CI) | 0.85 (0.53-1.38) | | 0.71 (0.46-1.07) | |

* Adjusted for age, sex, CHA2DS2-VAS score, Charlson score index, Frailty score, chronic renal failure
< 60 mL/min, prior major bleeding

IPTW: inverse probability of treatment weighting, SE: systemic embolism, CI: confidence interval, HR: hazard ratio, PS: propensity score, GI: gastro-intestinal
