# Supplementary material for: Effectiveness and safety of low-dose versus standard-dose rivaroxaban and apixaban in patients with atrial fibrillation
Source: PLoS One. 2022 Dec 1;17(12):e0277744. doi: 10.1371/journal.pone.0277744 (PMC9714756; doi:10.1371/journal.pone.0277744)
Supplement: Attachment — Submitted filename: Response to Reviewers.docx [file pone.0277744.s022.docx]

**Faculté de Pharmacie**

Université de Montréal

**February 22, 2022**

**Response to the Review Comments**

Many thanks for the excellent comments which improve the clarity of the manuscript and the robustness of the results.

***Reviewer #1:*** The manuscript has some significant flaws in design and execution which make it challenging to review:

**1)** The appropriate dosage of DOAC (Low/high) is determined by clinical characteristics, such as age and renal function:

**In response:** We appreciate the excellent comment. We clearly understand that dose of DOAC used in clinical practice is determined by patient characteristics such as, age and renal function. However, there is no head-to-head RCTs comparing individual DOACs, but indirect comparisons have been done using data from observational studies and DOACs pivotal RCTs (Verdecchia P et al., PlosOne 2014; Bai Y et al., Am J Cardiol 2017). According to the guideline of CHEST in 2018, there are DOAC recommendation for different subgroups of patients with atrial fibrillation (Lip GYH et al, Chest 2018). For instance, standard-dose dabigatran is recommended for patients with recurrent thrombosis events (Verdecchia P et al., PlosOne 2014), and apixaban is recommended for patients at high risk of gastrointestinal bleeding (Verdecchia P et al., PlosOne 2014; Bai Y et al., Am J Cardiol 2017). Although, the European guidelines maintained that DOAC dose selection should be dependent on age, renal function, weight and concomitant drug and BMI (Hindricks G et al., Eur Heart J 2021).

Moreover, a recent patient-level network meta-analyses of RCTs of DOACs in patients with atrial fibrillation reported that compared to lower-dose DOAC, patients randomized to standard-dose DOAC presented a lower risk of stroke/SE (HR 0.76 (0.68-0.86) and of composite efficacy outcome (HR 0.89 (0.82-0.96) (Carnicelli AP et al., Circulation 2022). This study presents some limitations such as, drug adherence was not taken into consideration and combining RCTs with different study drugs and doses for meta-analyses may not reveal the difference between outcomes that are specific each DOAC. Again, low-dose apixaban represented only 4% of the patients of ARISTOLE study, and low-dose rivaroxaban of ROCKET study was recommended only in patients with a creatinine clearance of 30 to 49 ml per minute) and seems to represent a low number of patients.

Given that the use of low-dose DOACs is more prevalent in clinical practice than expected and with more heterogeneous populations, and that the data from RCTs on its impact are limited, it is important to assess the effectiveness and safety of low-dose vs standard-dose DOACs used in real-world populations. We review the introduction to emphasize on this issue at Page 4, Para 1, as followed: ***A recent patient-level network meta-analyses of RCTs of DOACs versus warfarin in patients with AF reported results that compared to lower-dose DOAC, patients randomized to standard-dose DOAC presented a lower risk of stroke/SE (HR 0.76 (0.68-0.86) and of composite efficacy outcome (HR 0.89 (0.82-0.96). However, this study presents limitations such as, drug adherence was not taken into consideration and combining RCTs with different study drugs and doses for meta-analyses may not reveal the difference between outcomes that are specific each DOAC.(20)***

**2)** The study design ignores this issue completely- and to my understanding this is a major flaw. Ignoring the dosage labeling may create significant biases. It is impossible to estimate how the lower effectiveness is a result of a lower dose, or perhaps an inappropriate dose.

**In response:** First, as previously discussed, we suspected that the low-dose rivaroxaban versus standard-dose and the low-dose apixaban versus standard-dose apixaban may have different impacts on outcomes in heterogeneous populations of clinical practice. Several reasons supported the hypothesis: **1)** The estimated increased risk of stroke in the patient-level meta-analysis was represented with few cases of low-dose rivaroxaban and low-dose apixaban compared to those of dabigatran and edoxaban (Carnicelli AP et al., Circulation 2022); **2)** The pattern of prescribing is higher for rivaroxaban and apixaban than dabigatran or edoxaban (Perreault S et al Pharmacotherapy 2020); **3)** In the study of Testa et al., the thrombotic complications only occurred in AF patients primarily treated with low-dose DOAC and those with a high CHA_2_DS_2_-VASc score with a very low C-trough level (Testa S et al., Thromb Haemost 2018); and **4)** The risk profile of patients with atrial fibrillation in real-life studies is higher than those seen in RCTs, and the higher rates of major bleeding and mortality not attributable to thromboembolism in patients treated with apixaban 2.5 compared to 5 mg twice daily seem to be important causes to the higher than expected thromboembolic event rates in clinical (de Vries TAC et al, Thromb Haemost 2020)*.* We thus modified the introduction to emphasize on this issue at Page 4, Para 2, as followed: ***Given that the use of low-dose DOAC is more prevalent in clinical practice, and that close to 50% of patients who receive the low-dose apixaban do not meet the least two of three clinical characteristics,(21) it is important to assess the effectiveness and safety of low-dose vs standard-dose DOACs used in AF patients with similar baseline characteristics*** *(14, 15).*

Second, giving that there is no head-to-head comparisons between individual DOACs, we need to use observational study design which may be biased by confounding by indication. For instance, propensity score matching (PS-Match) and inverses probability of treatment weighting (IPTW) are used to address confounding by indication potentially relate to comparative effectiveness research, giving the lack of randomization in treatment allocation. Those methods challenge the properties of RCT by creating treatment and comparator with balanced distributions of patient characteristics. Both methods have different interpretations. In the case where there are an adequate sample size of comparators for matching to treated patients, PS-Match offers a more clear method such as, easy to understand and to explain. Moreover, PS-Match may also be more robust to misspecification of the propensity score than the IPTW method, where extreme weights can bias the estimation of the treatment effect. Although, IPTW has its advantages in retaining all eligible patients, which may be preferred if there are limitations in terms of sample size, (Allan V et al., J Comp Eff Res 2020).

Thus, we judge that both methods can be applicable for estimating an average treatment effect (IPTW) and the treatment (PS-Match). However, PS-Match may limit the representativeness of the study population and generalizability of the overall study findings. We are now providing data on PS-Matching 1:1. The baseline characteristics after PS-Matching (1:1) are presented at S15 Table. We presented the comparative effectiveness and safety outcomes in PS-matched cohorts on-Treatment (UT) and intent-to-treat (ITT) at S16 Table*.* We modified the methods section at page 8, Para 4 as followed for the sensitivity analyses: ***Third, we used 1:1 PS-matching without replacement and match on the logit of the PS using calipers of width equal to 0.2 of the standard deviation of the logit of the PS.(43) We used a multivariable logistic regression model to estimate the PS of the patients receiving low vs standard dose DOACs, based on baseline characteristics (Table S15).***

We also add at Page 24, Para 2, the results of 1:1 PS-Match as followed: ***We also assess a 1:1 PS matching without replacement on the outcomes for UT and ITT analyses. Pairwise comparisons of low-dose and standard-dose rivaroxaban or low-dose and standard-dose apixaban matched for sociodemographic and clinical characteristics are shown in Table S15. Similar results were observed as those observed using IPTW method (Table S16).*** ***Moreover, the results were also similar to those using adjusted raw data for UT analyses (Table S17).***

We also add limits in the discussion for advantage and limits of PS-Match and IPTW at Page 27, Para 2, as followed: ***‘Second, PS-Match and IPTW*** ***method have different interpretations. In the case where there are an adequate sample size of comparators for matching to treated patients, PS-Match offers a more clear method. PS-Match may also be more robust to misspecification of the propensity score than the IPTW method, where extreme weights can bias the estimation of the treatment effect. Although, IPTW has its advantages in retaining all eligible patients, which may be preferred if there are limitations in terms of sample size where PS-Match may limit the representativeness of the study population and generalizability of the overall study findings (22).***

Finally, we also modified the discussion at Page 26, Para 1 as followed: ***Again, close to 50% of patients who receive the low-dose apixaban do not meet the least two of three clinical characteristics [21]. The higher risk profile of AF patients in real-life studies than those seen in RCTs, and the higher rates of major bleeding and mortality not attributable to thromboembolism in AF patients treated with apixaban 2.5 compared to 5 mg twice daily seem to be important causes to the higher than expected thromboembolic event rates in clinical [21].***

**3.1)** Even if we ignore this flaw, the use of IPTW is not sufficient and not sure it is appropriate for the study objective. For example, the age of the low dose apixaban before IPTW is 10 years higher than the high dose- suggesting that the low dose maybe a result of the age of the patients. I would expect to see the real (raw) number of events in each group. The data is disclosed in Table S8, is after IPTW, and does not provide this info.

**In response:** We provide the comparative effectiveness and safety outcomes on under treatment (UT) cohort from raw data, IPTW method and PS-Match 1:1 at S17 Table. The results of adjusted raw data were also in accord with IPTW and PS-Match methods. We add at Page 24, Para 2 as followed: ***Moreover, the results were also similar to those using adjusted raw data for UT analyses (Table S17).***

**3.2)** At the least, I would expect using an alternative method for assessing effectiveness to validate the IPTW modeling. The abundance of data should be enable these analyses.

**In response**: Like previously discussed, we provide 1:1 PS-Match analysis as sensitivity analyses for under treatment and intent to treat in Table S16. The results were in accord with IPTW method. Based on the sample size, we judge that both methods can be applicable and estimating an average treatment effect (IPTW) and the treatment (PS-Match) but the PS-Match results may limit the representativeness of the study population and generalizability of the overall study findings.

In addition, we provide the comparative effectiveness and safety outcomes on under treatment (UT) cohort from raw data, PS-Match 1:1 and IPTW method in S17 Table.

***Reviewer #2***: Perreault et al used Canadian administrative databases to study the effectiveness and safety of low dose vs high dose rivaroxaban and apixaban in patients with atrial fibrillation. The authors found no clear differences in effect or safety comparing high with low dose of either drug. I have a number of questions/comments regarding the study’s methods that should be answered before evaluation. Essentially, I would like to see a clearer description of the Methods section.

**In response:** As previously presented, we find difference. For instance, No significant difference was observed in the incidence of comparative stroke/SE and MB between low-dose and standard-dose rivaroxaban, except for the risk of acute myocardial infarction, which was increased with the low dose in the UT analysis. For apixaban, no difference was found in the bleeding rates, but the risk of stroke/SE (HR: 1.95; 1.38–2.76) and death (HR: 1.99; 95% CI: 1.46–2.70) were greater in the low-dose group than in the standard-dose group.

**1.** The authors used data from the “Med-Echo” databases. For authors should include a description of these data sources as most readers will be unaware of the infrastructure of these data sources. A few additional lines could be added to the main text, with the rest included in the Appendix. As of now, no description is present, even in the Appendix.

**In response**: Many thanks for the comment, this clarifies the data source. In the S1 Table, we included informations reletad to the data source: The cohort from the Med-Echo administrative databases, which store data on hospital discharges, medical services, and public drug plans, managed by the Régie de l’Assurance Maladie du Québec (RAMQ).(1-4) The databases were linked using encrypted health insurance numbers. The information from these databases provide a comprehensive picture of the status of hospital admissions.

We have also added more detail in the appendix Table S1, and its validity. Data were collected from the “Régie de l’Assurance Maladie du Québec” (RAMQ) and Med-Echo databases, which administer public healthcare insurance programs in the province of Québec, Canada. The Med-Echo database gathers information on acute care hospitalizations, such as date of admission, length of stay, primary and up to 15 secondary diagnoses. The RAMQ data were extracted from three databases. The beneficiary database lists age, gender, socioeconomic status, and date of death. The medical services file contains claims for all inpatient and ambulatory services and diagnostic codes are classified according to the International Classification of Diseases, 9^th^ revision (ICD-10). All surgical procedures codes follow the Canadian classification of diagnostic, therapeutic and surgical procedures.^5^ The pharmaceutical database provides data on delivered medication in community pharmacies such as the date of filling, name of the drug, dose, quantity, dosage form, and duration of therapy. The RAMQ covers all Quebec residents for the cost of physician visits, hospitalizations and procedures, and 94% of Quebec citizens aged 65 and older for the drug plan.^3,6^

1. Tamblyn R, Lavoie G, Petrella L, Monette J. The use of prescription claims databases in pharmacoepidemiological research: the accuracy and comprehensiveness of the prescription claims database in Quebec. J Clin Epidemiol. 1995;48(8):999-1009.
2. Eguale T, Winslade N, Hanley JA, Buckeridge DL, Tamblyn R. Enhancing pharmacosurveillance with systematic collection of treatment indication in electronic prescribing: a validation study in Canada. Drug Saf. 2010;33(7):559-67.
3. Wilchesky M, Tamblyn RM, Huang A. Validation of diagnostic codes within medical services claims. J Clin Epidemiol. 2004;57(2):131-41.
4. Tamblyn R, Reid T, Mayo N, McLeod P, Churchill-Smith M. Using medical services claims to assess injuries in the elderly: sensitivity of diagnostic and procedure codes for injury ascertainment. J Clin Epidemiol. 2000;53(2):183-94.
5. Régie de l'assurance maladie du Québec. Rapport annuel de gestion 2005-2006. Quebec: Régie de l'assurance maladie du Québec; 2006.
6. Tamblyn R, Lavoie G, Petrella L, Monette J. The use of prescription claims databases in pharmacoepidemiological research: the accuracy and comprehensiveness of the prescription claims database in Quebec. *J Clin Epidemiol.* Aug 1995;48(8):999-1009.

**2.** The authors should make the cohort entry date (or index date) more explicit for the reader. I suspect this data to be the date of drug filling, but it is not clear. Also, please be consistent regarding terminology (cohort entry vs index date). Relatedly, it would be nice if the authors included a study diagram, which makes it easier for readers to understand the study (see <https://doi.org/10.7326/M18-3079>)

**In response:** We changed the flow chart to improve the understanding for low-dose and standard-dose definition. As shown at Page 5, Para 3, we modified the text as followed: We identified patients who filled a new prescription for rivaroxaban (**low-dose 15 or standard-dose 20 mg once daily)** or apixaban (**low-dose 2.5 or standard-dose 5.0 mg twice daily)** within a year after hospital discharge. The patients were new users, defined as no exposure to any OAC one year prior to the claim index date. Eligible patients were required to have pharmacy coverage for at least 12 months and had been continually enrolled in an insurance drug plan for at least one year **before the claim index date which defined the cohort entry**.

**3**. The authors used a quite long exposure assessment window. Can the authors provide the median time to initiation by exposure group? What happened if an AF patient died (or experienced a stroke) before DOAC initiation?

**In response:** Claims was filled ≥ 80% and 95.6% in the next 7 days and 90 days following discharge, respectively. The median time to DOAC initiation after AF hospitalization was 0 day (i.e. a majority of patients were initiated to DOAC the day of AF hospitalization discharge). They need to be alive for receiving the first index claim of OAC which defined the date of cohort entry.

**4.** Please describe the exposures (ie. low vs high) more clearly. Which specific drugs were classified as low and which as high?

**In response.** We identified patients who filled a new prescription for rivaroxaban (low-dose 15 or standard-dose 20 mg once daily) or apixaban (low-dose 2.5 or standard-dose 5.0 mg twice daily) within a year after hospital discharge. We changed the flow chart to improve the understanding, and we modified the Page 5, Para 3 as followed*:* ***We identified patients who filled a new prescription for rivaroxaban (low-dose 15 or standard-dose 20 mg once daily) or apixaban (low-dose 2.5 or standard-dose 5.0 mg twice daily) within a year after hospital discharge.***

**5.** Please provide a rationale for the exclusion criteria.

**In response:** We excluded patients who had undergone cardiac valvular replacement or valvular procedures within five years of cohort entry. The reasons are related to the guideline recommendations. We also excluded patients who were diagnosed with end-stage chronic kidney disease, those who had undergone kidney transplantation, those who were on dialysis in a 3-year period prior to cohort entry, where DOACs is contraindicated.

For those who were diagnosed with deep vein thrombosis or who had undergone orthopedic surgery within three months prior to cohort entry, the indications of OAC is for the short period instead of a planned long-term treatment.

Finally, we excluded patients with a coagulation deficiency or those who had undergone certain medical procedures, including stent, coronary artery bypass grafting, cerebrovascular interventions, or cardiac device implantation within three months of cohort entry. The reasons for excluding those patients for coagulation deficiency or with recent procedures that might influence OAC treatment.

**6.** Why was IPTW used over other weighting methods? What question was trying to be answered?

**In response:** See prior responses and comments.

**7.** The authors include a range of covariates in their model, but the covariates are not clearly described before Table.

**Response**: The covariates are described in the section of ‘Patient demographics and clinical characteristics’. Those covariates are similar to several papers on comparative effectiveness and safety (Allan V et al., J Comp. Effec. Res. 2020; Rahme E et al., Thromb. Res. 2021.)

**6.** PLOS authors have the option to publish the peer review history of their article ([what does this mean?](https://journals.plos.org/plosone/s/editorial-and-peer-review-process#loc-peer-review-history)). If published, this will include your full peer review and any attached files.

**In response:** No problem

Sincerely yours,

_____________________________________

Sylvie Perreault, BPharm, PhD

Professeure Titulaire

Chaire Sanofi Canada sur l’usage optimal des médicaments

Faculté de pharmacie

Université de Montréal

Tél : (514) 343-6111 poste 3149

Télécopieur : (514) 343-6120

Sylvie.perreault@umontreal.ca
