# Supplementary material for: Effectiveness and safety of low-dose versus standard-dose rivaroxaban and apixaban in patients with atrial fibrillation
Source: PLoS One. 2022 Dec 1;17(12):e0277744. doi: 10.1371/journal.pone.0277744 (PMC9714756; doi:10.1371/journal.pone.0277744)
Supplement: Attachment — Submitted filename: 2022-10-26.Response.Reviewer.PONE.docx [file pone.0277744.s023.docx]

**PONE-D-21-29130R1**

Effectiveness and safety of low-dose versus standard-dose rivaroxaban and apixaban in patients with atrial fibrillation

**Please answer to the issues raised by the reviewer. In case the issue cannot be solved by a reanalysis of the data, the limitation should be clearly stated in the revised manuscript**.

Many thanks for the excellent comments which improve the clarity of the manuscript.

**Comments to the Reviewer 1**

**Reviewer #1**: In short, the response to the effectiveness evaluation methods (my third comment) is satisfactory.
However, the answers to my first 2 comments (Although very details) do not answer my very basic and important comment regarding the appropriateness of dosage, according to FDA dosage labels for each drug. I do not think that this issue can be answered only by the literature, but has to be analyzed by the data. Even if the authors disagree to make it the primary analysis, it should be done as a secondary or sensitivity analysis.

**In response:** As reported in the limits section, this is an observational study using administrative data and may be subject to confounding bias by unadjusted factors (e.g., AF severity, blood pressure control, exact estimated glomerular filtration rate, international normalized ratio, body weight, and over-the-counter prescriptions) or by a residual channeling bias.

We are not able to provide the appropriateness of the prescription without having the exact weight and the exact estimated glomerular filtration rate. But, for chronic kidney disease, we have a validated algorithm for the stage of chronic kidney disease. The category of estimated glomerular filtration rate was estimated with an algorithm based on diagnosis code, drug use, and nephrologist visits from administrative databases that shown to be valid when compared with medical chart reviews in older adults (Roy L et al. Can J Kidney Health Dis 2020). The algorithm used for eGFR definition had a positive predictive value ranging from 94.5% to 97.7% (Roy L et al 2020). **We modified the method section at Page 7, line 140-144 as followed:** **The category of chronic kidney disease was assessed using the estimated glomerular filtration rate using an algorithm based on diagnosis code, drug use, and nephrologist visits from administrative databases that shown to be valid when compared with medical chart reviews in older adults. The algorithm used for eGFR definition had a positive predictive value ranging from 94.5% to 97.7%**

In order to assess the robustness of the results we provided several sensitivity analyses to support our hypothesis that given that the use of low-dose DOAC is more prevalent in clinical practice, and that close to 50% of patients who receive the low-dose apixaban do not meet the least two of three clinical characteristics (de Vries TAC et al. 2020), it is important to assess the effectiveness and safety of low-dose vs standard-dose DOACs used in AF patients with similar baseline characteristics (Maura G et al, 2017; Perreault S et al 2022). Due to the lack of randomization in treatment assignment, we used an inverse probability treatment weighting (IPTW) approach to minimize the impact of confounding by indication in representative cohorts of AF patients to assess the effectiveness and safety of low-dose vs standard-dose rivaroxaban and apixaban (Allan V et al, 2020).

**We modified the limit section at Page 26, Para 307-309, as followed****:** **Second, we are not able to provide the appropriateness of the prescription without having the exact weight and the exact estimated glomerular filtration rate but in order to assess the robustness of the results, we ran several sensitivity analyses.**

**Reviewer #2**: The authors have adequately addressed my comments related to the methods section. I have no further concerns.
